# Protocol for a randomized controlled trial to evaluate a year-long (NICU-to-home) evidence-based, high dose physical therapy intervention in infants at risk of neuromotor delay

**DOI:** 10.1371/journal.pone.0291408

**Published:** 2023-09-19

**Authors:** Weiyang Deng, Sofia Anastasopoulos, Raye-Ann deRegnier, Nicole Pouppirt, Ann K. Barlow, Cheryl Patrick, Megan K. O’Brien, Sarah Babula, Theresa Sukal-Moulton, Colleen Peyton, Catherine Morgan, John A. Rogers, Richard L. Lieber, Arun Jayaraman

**Affiliations:** 1 Shirley Ryan AbilityLab, Chicago, Illinois, United States of America; 2 Division of Neonatology, Ann & Robert H. Lurie Children’s Hospital of Chicago, Chicago, Illinois, United States of America; 3 Department of Pediatrics (Neonatology), Feinberg School of Medicine, Northwestern University, Chicago, Illinois, United States of America; 4 Division of Rehabilitative Services, Ann & Robert H. Lurie Children’s Hospital of Chicago, Chicago, Illinois, United States of America; 5 Department of Physical Medicine & Rehabilitation, Feinberg School of Medicine, Northwestern Medicine, Chicago, IL, United States of America; 6 Pathways.org, Shirley Ryan AbilityLab, Chicago, Illinois, United States of America; 7 Department of Physical Therapy and Human Movement Sciences, Feinberg School of Medicine, Northwestern University, Chicago, IL, United States of America; 8 Department of Pediatrics, Feinberg School of Medicine, Northwestern University, Chicago, IL, United States of America; 9 Cerebral Palsy Alliance Research Institute, Discipline of Child and Adolescent Health, The University of Sydney, Sydney, New South Wales, Australia; 10 Department of Biomedical Engineering, Querrey Simpson Institute for Bioelectronics, Northwestern University, Evanston, Illinois, United States of America; 11 Departments of Materials Science and Engineering, Chemistry, Mechanical Engineering, Electrical Engineering and Computer Science, Northwestern University, Evanston, Illinois, United States of America; 12 Department of Neurological Surgery, Northwestern University Feinberg School of Medicine, Northwestern University, Chicago, Illinois, United States of America; 13 Department of Neuroscience, Feinberg School of Medicine, Northwestern University, Chicago, Illinois, United States of America; 14 Jessie Brown Jr., Hines V.A. Medical Center, Hines, Illinois, United States of America; 15 Department of Medical Social Sciences, Feinberg School of Medicine, Northwestern University, Chicago, Illinois, United States of America; University Medical Centre Ljubljana (UMCL) / Faculty of Medicine, University Ljubljana (FM,UL), SLOVENIA

## Abstract

**Introduction:**

Developmental disabilities and neuromotor delay adversely affect long-term neuromuscular function and quality of life. Current evidence suggests that early therapeutic intervention reduces the severity of motor delay by harnessing neuroplastic potential during infancy. To date, most early therapeutic intervention trials are of limited duration and do not begin soon after birth and thus do not take full advantage of early neuroplasticity. The Corbett Ryan–Northwestern–Shirley Ryan AbilityLab–Lurie Children’s Infant Early Detection, Intervention and Prevention Project (Project Corbett Ryan) is a multi-site longitudinal randomized controlled trial to evaluate the efficacy of an evidence-based physical therapy intervention initiated in the neonatal intensive care unit (NICU) and continuing to 12 months of age (corrected when applicable). The study integrates five key principles: active learning, environmental enrichment, caregiver engagement, a strengths-based approach, and high dosage (ClinicalTrials.gov identifier NCT05568264).

**Methods:**

We will recruit 192 infants at risk for neuromotor delay who were admitted to the NICU. Infants will be randomized to either a standard-of-care group or an intervention group; infants in both groups will have access to standard-of-care services. The intervention is initiated in the NICU and continues in the infant’s home until 12 months of age. Participants will receive twice-weekly physical therapy sessions and caregiver-guided daily activities, assigned by the therapist, targeting collaboratively identified goals. We will use various standardized clinical assessments (General Movement Assessment; Bayley Scales of Infant and Toddler Development, 4th Edition (Bayley-4); Test of Infant Motor Performance; Pediatric Quality of Life Inventory Family Impact Module; Alberta Infant Motor Scale; Neurological, Sensory, Motor, Developmental Assessment; Hammersmith Infant Neurological Examination) as well as novel technology-based tools (wearable sensors, video-based pose estimation) to evaluate neuromotor status and development throughout the course of the study. The primary outcome is the Bayley-4 motor score at 12 months; we will compare scores in infants receiving the intervention *vs*. standard-of-care therapy.

## 1. Introduction

More than 17% of children and adolescents born in the United States have developmental impairments [1] that may lead to long-term disability and potential adverse socio-economic and health consequences [2]. Furthermore, caregivers of infants at risk of developmental disabilities face significant long-term psychological, physical, and financial stress [3]. Various intervention strategies have been explored to reduce the impact of developmental disabilities in infants. Current evidence suggests that parent-provided interventions are more beneficial than purely clinician-focused interventions [4–11], as caregivers are the most motivating ‘toy’/therapeutic interaction for an infant [12, 13]. In addition, ensuring active infant participation is necessary to maximize brain neuroplasticity [12, 14–17], resulting in better cognitive and motor outcomes compared to passive interventions alone [18–24]. This active learning process is further facilitated by an enriched environment to facilitate infants’ exploratory learning behavior [25], providing the “just right challenge” [26] to promote brain recovery and optimal developmental outcome [27–34]. Ideally, to improve these intervention outcomes, not only do infants need to be motivated during the learning process, caregivers also must be encouraged to think optimistically about their child’s potential, to focus on opportunities for growth [35–38], and to be confident and engaging during interactions with their infant [39–42]. Finally, higher dose interventions are typically more effective compared to lower dose interventions in promoting motor development and positive neuronal adaptations [14, 16, 28, 43–45].

Current evidence also encourages early initiation of therapeutic intervention to mitigate neuromotor conditions and improve cognitive and motor skills for infants at risk of neuromotor delay [8, 9, 43, 46–50]. Research suggests that an infant’s brain has greater potential for neural plasticity early in life, which may enable adaptation and reorganization after perinatal injury [8, 48–50]. An early onset intervention (e.g., beginning in the neonatal intensive care unit, (NICU) may result in greater restoration of neural circuitry and functional skill compared to an intervention initiated later in life [8, 38, 43, 48]. However, although infants at risk of developmental disabilities may receive physical therapy in the NICU, access to further standard-of-care therapy services is often delayed until well after NICU discharge [45, 51–57]. Despite the wealth of evidence presented supporting early therapeutic intervention with highly engaged caregivers, no large-scale clinical trial has rigorously evaluated the effects of an early onset, strategic, long-term continuum of care intervention on neuromotor development. Most completed or ongoing clinical trials of early physical therapy-based interventions have a duration of less than three months and/or are focused on specific high-risk populations [30, 43, 47, 58–60], thereby missing infants who may go on to develop cerebral palsy but were not initially identified as high risk [61, 62].

Based on the cumulative evidence presented above for effective intervention strategies and the benefits of early therapeutic intervention, we designed “Project Corbett Ryan”—a randomized controlled trial (RCT) of a high-dosage, caregiver-led physical therapy intervention beginning in the NICU and continuing to 12 months of age (corrected, as applicable), with longitudinal follow up for up to 24 months of age. Project Corbett Ryan expands upon the state-of-the-art Caregiver engagement, Active Learning, Environmental enrichment, and Strengths-based (CARES) theoretical framework to maximize the neuroplastic potential of infants at risk for neuromotor delay [39]. The intervention involves therapy activities that are tailored to each participant and designed based on five key principles: caregiver engagement, active learning, environmental enrichment, a strengths-based approach, and high dosage.

The primary aim of this study is to evaluate the efficacy (i.e., how well this intervention is performed under ideal and controlled circumstances, where all participants received all intervention sessions) and effectiveness (i.e., how well this intervention is performed in a real world situation, where participants receive fewer intervention sessions) of this intervention to improve motor function and reduce severity of motor delay at 12 months of age. We hypothesize that the Bayley Scales of Infant and Toddler Development assessment 4^th^ edition (Bayley-4) motor score at 12 months of age will average 8-points higher (0.5 standard deviation)—a clinically meaningful difference [63]—in the intervention group compared to the standard-of-care group.

The secondary aim is to evaluate the early effects of this intervention on motor function and severity of motor delay. We hypothesize that infants in the intervention group will demonstrate statistically significant improved motor function and reduced motor delay compared to the standard of care group as early as 3 months of age.

Finally, we will quantify the dose-response relationship between motor function at 12-months of age and intervention dose (defined as the number of therapist sessions and/or total hours of caregiver-provided intervention during the 12-month intervention period). We hypothesize that a higher intervention dose will correlate with higher Bayley-4 motor scores at 12-months of age.

## 2. Materials and methods

**Fig 1:**
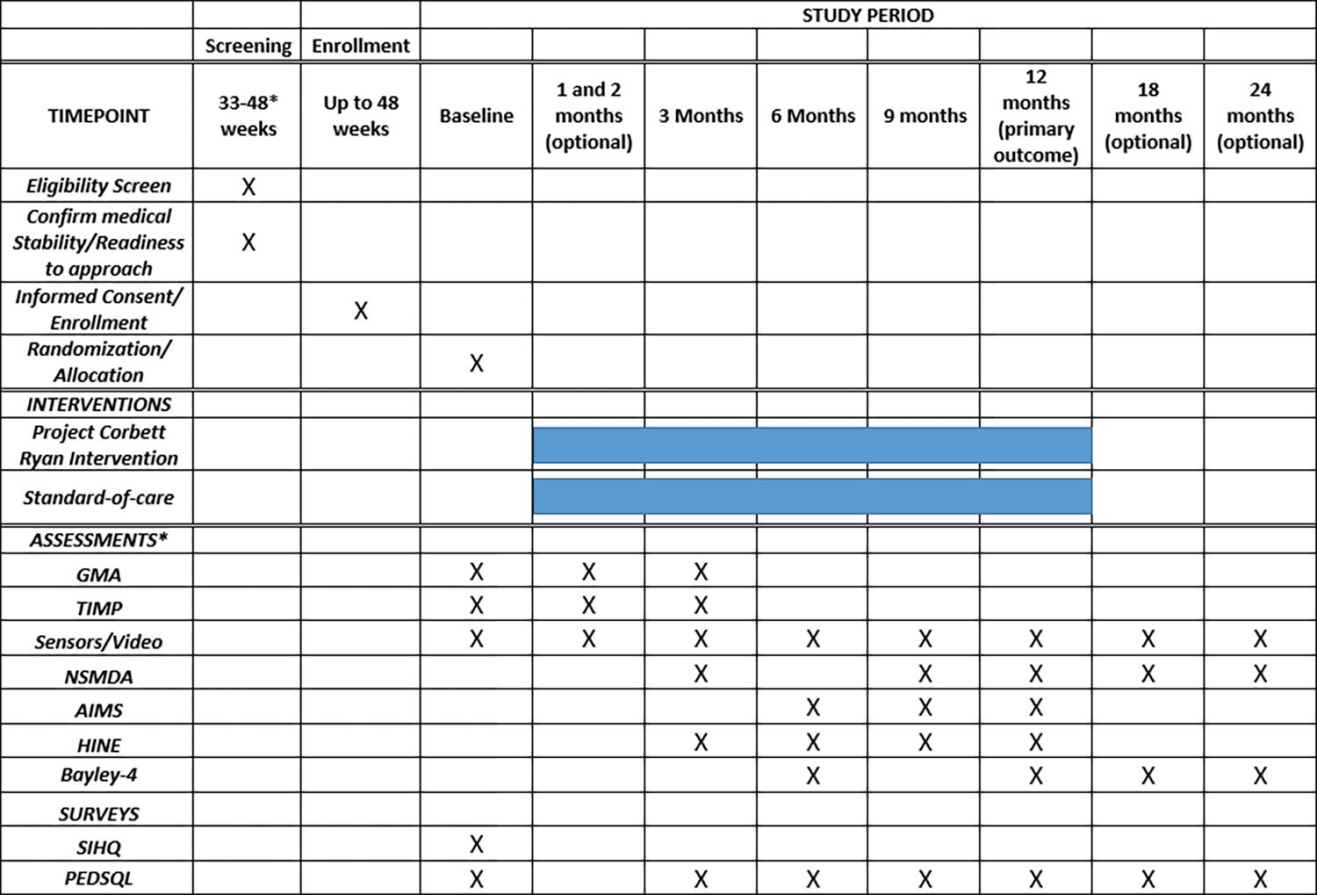
SPIRIT schedule of enrollment, intervention, and assessments. *Assessments are conducted at chronological age or corrected age, as appropriate.

GMA: General Movement Assessment; TIMP: Test of Infant Motor Performance; HINE: Hammersmith Infant Neurological Examination; NSMDA: Neurological, Sensory, Motor, Developmental Assessment; Bayley-4: AIMS: Alberta Infant Motor Scale; Bayley Scales of Infant and Toddler Development, 4^th^ edition; PEDI-CAT: Pediatric Evaluation of Disability Inventory; WIDEA-FS: Warner Initial Developmental Evaluation of Adaptive and Functional Skills; SIHQ–social influencers of health questionnaire; PedsQL: Pediatric Quality of Life Inventory Family Impact Module.

Project Corbett Ryan is a two-arm randomized controlled trial that evaluates a one-year-long physical therapy intervention in infants admitted to a level III or IV NICU from three recruitment sites in the Chicagoland area. The trial is conducted under a single reliance agreement and reviewed and approved by the Institutional Review Board (The Ann & Robert H. Lurie Children’s Hospital of Chicago Institutional Review Board). All reliance and data use agreements were executed prior to enrollment at each site to ensure participant protection. Any necessary modifications to study procedures will first be approved by the IRB of record and communicated to all parties as required. Caregivers will provide written informed consent for their infant’s participation in the study. The study was launched in October 2022 and is currently recruiting participants; it is scheduled to end in December 2026. Fig 1 illustrates the SPIRIT schedule for the study, including enrollment, baseline measures and study arm allocation, intervention administration, and assessments [64, 65].

### 2.1 Participants

We will enroll 192 infants. Eligible infants will demonstrate a risk for neuromotor delay and qualify for follow-up at the NICU developmental follow-up clinic at each recruitment site. Infants will be ineligible if their condition or diagnosis prevents participation in the intervention, or clinical/sensor-based assessments. Detailed eligibility criteria are listed in Table 1.

**Table 1:** Participant inclusion and exclusion criteria.

Category:	Description:
**Inclusion Criteria**	
Age of Enrollment	Participants must be consented by 48 weeks PMA.
Medical Diagnosis	NICU admission and diagnosis that would qualify for monitoring in the NICU developmental follow-up clinic and/or Early Intervention Services due to any of the following: • Birth weight <1500 grams • Disorder of the Central Nervous System, including: ○ Brain Injury (including but not limited to extra-axial hemorrhage, any grade intraventricular or intraparenchymal hemorrhage, stroke, hypoxic-ischemic encephalopathy (HIE), and meningitis) ○ At risk for HIE (10-minute APGAR <7 and either a pH or <7.15 or base deficit ≥ 12) ○ Brain Developmental Abnormality ○ Cramped Synchronous movements on the General Movements Assessment (GMA) at term-post menstrual age • Bronchopulmonary dysplasia defined as a need for respiratory support at 36 weeks PMA in an infant born <32 weeks gestation
Medical Stability	Participants will be medically stable and able to start the intervention between 34–48 weeks PMA
**Exclusion Criteria**	
Medical Diagnosis	A medical diagnosis that would impact participation in the intervention, sensor placement, or clinical assessments including any of the following: • Open wounds or skin conditions impacting sensor placement • Immune deficiencies requiring protective isolation • Limb reduction deficits • Bleeding disorders or the need for ongoing anticoagulation • Known visual impairment at time of enrollment • Infants followed in a different developmental clinic (including but not limited to meningomyelocele and related conditions/trisomy 21) • Infants on palliative or hospice care (for life-limiting conditions including but not limited to trisomy 18, 13) • Any other condition that would preclude study participation, based on discretion of study neonatologist
Language	No English-speaking caregivers available
Protective Custody	The infant is in the Department of Children and Family Services’ custody
Concurrent Study Participation	The infant is enrolled in a concurrent randomized trial with developmental outcome variables.

### 2.2 Screening and recruitment

Infants are screened through an electronic medical record (EMR) review starting at 33–34 weeks post-menstrual age (PMA). Prior to consent, the infant’s eligibility for participation, readiness to approach, based on approval of primary medical team, and medical stability are reviewed. Consent will be obtained by approved study team members at each study site. Consent is initiated in the NICU but may be completed after discharge. Infants may be consented up to 48 weeks PMA.

### 2.3 Risk stratification and randomization

Once an infant is enrolled in the study, their risk of neuromotor delay (mild or moderate-severe) is determined by a study neonatologist at each recruitment site [66–74]. The criteria used to make this determination are shown in Table 2. Randomization to the intervention group or the standard-of-care group is stratified based on risk of neuromotor delay and recruitment site and completed within a REDCap project [75, 76] using a randomized complete block design. The two study groups (intervention and standard of care) are assigned randomly within these blocks. Multiple births, where two infants are eligible for participation, are treated as a unit and randomized using a separate REDCap project in order to allocate them to be placed in the same study group while also accounting for each infant’s individual risk of motor delay and recruitment site. Randomization matrices were created by the study statistician.

**Table 2:** Criteria used to stratify an infant’s risk of neuromotor delay.

Risk for Neuromotor Delay:	Description:
**Mild**	As indicated by any of the following: • At risk for HIE—10-minute Apgar <7 and either a pH <7.15 or a base deficit ≥ 12 • Clinical HIE or meningitis with normal MRI or no seizures • Subdural, subarachnoid, or subgaleal hemorrhage with otherwise normal MRI and no seizures • Mild abnormalities on cranial ultrasound or MRI (including, but not limited to, non-cystic periventricular leukomalacia (PVL) or white matter injury) • BW <1500 grams with grade 0, 1, or 2 intraventricular hemorrhage (IVH) • No Bronchopulmonary Dysplasia (BPD) or Jensen Grade 1 BPD (nasal cannula (NC) ≤ 2 liters per minute (LPM) at 36 weeks)
**Moderate-severe**	As indicated by any of the following: • Clinical HIE diagnosis or meningitis with abnormal MRI or seizures • Grade 3 or 4 hemorrhage • Cystic PVL • Ventriculoperitoneal shunt • Stroke • Cramped-Synchronous movements on at term-post menstrual age GMA • BPD (respiratory support of > 2 LPM NC at 36 weeks) • Brain developmental abnormality (hydrocephalus, microcephaly, cortical dysgenesis) • Other surgical or medical conditions likely to increase the risk of neuromotor delay (based on clinical judgment)

Intervention physical therapists, project coordinators, sensor data collection team members, and study investigators will be aware of the infant’s study group allocation. Clinicians completing or scoring assessments and individuals responsible for sensor data analysis will be blinded to group allocation. Given the structure and scheduling system of the NICU, blinding clinical assessors in that setting may not always be feasible.

### 2.4 The intervention

Expanding upon the CARES framework [39], this intervention integrates the five principles of active learning, environmental enrichment, caregiver engagement, a strengths-based approach, and high dosage (Table 3). It will be initiated in the NICU, when possible, and continue until infants reach 12 months of age. Throughout the intervention, therapists will select activities from an Activity Playbook. All activities are designed and administered using these five principles. The therapist will select the activities tailored to the infant and new activities will be selected as the infant progresses. For the purposes of this paper, age in months refers to chronological age (for infants born at or after 37 weeks gestation) or corrected age (for infants born prior to 37 weeks gestation)

**Table 3:** Study key principles and strategy descriptions.

Key Principle	Definition	Implementation Strategies
**Active Learning**	Self-initiated, goal-directed movements encouraging the infant to explore their environment, thereby driving neuroplastic changes.	• Maximize participation with a motivating stimulus • Utilize the “Wait, Show, Help, Stop” strategy for task scaffolding • Support with the least amount of manual guidance • Encourage movement variability • Promote trial-and-error practice
**Environmental Enrichment**	Utilize naturally occurring resources within the infant’s surrounding environment and modify the task difficulty (cognitive or physical) to maximize opportunities for active learning.	• Utilize the “Just-Right Challenge” • Encourage social and object exploration • Modify the environment or cognitive demand of a task • Incorporate activities into the infant’s daily routine
**Caregiver Engagement**	Caregivers are identified as equal partners and encouraged to take an active, lead role in intervention delivery.	• Motivational interviewing • Caregiver coaching model inclusive of problem solving, reflection, practice, and feedback. • Collaborative goal setting • Caregivers are identified as equal partners in providing the intervention
**Strength-Based Approach**	A communication strategy that emphasizes the positive attributes of both the caregiver and the child. It establishes clear communication about areas of growth in a positive, capacity-building manner.	• Encourage problem-solving and decision-making • Collaborative goal setting • Reframing • Positive self-talk
**High Dosage**	Includes the number of intervention sessions and the amount of time the infant is engaged in activities targeting their motor goals.	• Evaluate facilitators and barriers to participation • Maximize opportunities for active learning throughout the infant’s day • Allow virtual intervention sessions, based on the unique characteristics of the infant and family • Structure both caregiver-led and child-led activities targeting distinct motor goals

#### 2.4.1 NICU

Infants will receive twice weekly intervention sessions lasting 40–60 minutes. Session length may be modified depending on the infant’s tolerance and family needs. Caregivers will be asked to complete Activity Playbook recommendations for at least 20 minutes per day, 5 days per week and to record the time spent on these activities using a written or electronic log. Activity playbook recommendations are based on goals collaboratively identified by the therapist and caregivers and progressed based on the infant’s functional ability.

The NICU intervention focuses on building caregiver competency in infant signal and stress cue management, maximizing and supporting caregiver confidence in infant interactions, introducing variable positions to promote caregiver engagement and infant tolerance to movement, and promoting active learning in different functional positions.

Collaborative goal setting will be completed between the therapist and caregivers; goals will target state regulation, gaze stabilization and tracking, head and trunk control, and early reaching. Additionally, early weight-bearing activities are included in the NICU intervention.

Prior to NICU discharge, we complete a transition visit that includes the caregiver(s), NICU therapist, and home therapist. If this is not feasible, home therapists are provided a summary of any NICU intervention sessions completed as well as any information collected as part of an EMR review. The first home intervention session will be scheduled within two weeks of NICU discharge.

#### 2.4.2 Home (up to 3 months)

Infants will receive twice-weekly intervention sessions lasting approximately 60 minutes, and therapists will continue to assign activity playbook recommendations to be completed for at least 20 minutes per day, 5 days per week. As in the NICU, activities are selected based on the infant’s current functional abilities and collaboratively identified goals. Due to the variability of infants’ age of enrollment and length of NICU stay, the intervention focus and goals may overlap with those from the NICU.

#### 2.4.3 Home (3 to 12 months)

Infants will receive twice weekly intervention sessions lasting approximately 60 minutes. Activity playbook dosage will be progressively increased to 60 minutes per day, 7 days per week. Recommendations may be divided into caregiver-led or child-led activities.

Intervention sessions during this time will focus on intentional, goal-directed movements that promote the infant’s ability to explore their environment. Collaboratively identified goals will target gaze stabilization and tracking, reaching and grasping, floor mobility, and exploration in different positions (transitions, sitting, standing, pre-gait/gait). Components of these goal areas are often intertwined; however, therapists will emphasize those aspects that best address the infant’s functional level and goals. Task structure will maximize the infant’s contribution and ensure that the activities are motivating and repeatable. Priority will be given to infant-generated movement strategies, with the therapist providing the least amount of manual guidance necessary to accomplish this goal, and less focus on achieving a normalized movement pattern. However, infant movement patterns may be modified if there is a lack of variability, if they limit the infant’s ability to explore the environment, or if there is a concern for injury. Additionally, if the caregiver or the therapist observes signs of neurologic injury (e.g., hemiplegia) or a consistent side preference, intervention sessions may be structured to address those concerns. For example, if there is a concern for hemiplegia, sessions will include targeted practice (gross and fine motor) using the affected side. Additional targeted therapies indicated for conditions such as torticollis or hemiplegia are provided outside of the study.

#### 2.4.4 Mode of session delivery

The primary mode of physical therapy delivery will be through in-person sessions. However, as needed, (e.g., due to illness) intervention sessions may be adjusted from in-person to virtual to ensure that infants receive the target intervention dosage. We will try to assign the same physical therapist for an infant, as schedules allow. However, depending on staff availability, different physical therapists may conduct intervention sessions with a given family. A detailed staff handoff procedure will be performed prior to the session in cases like this.

#### 2.4.5 Toy kits

**Fig 2:**
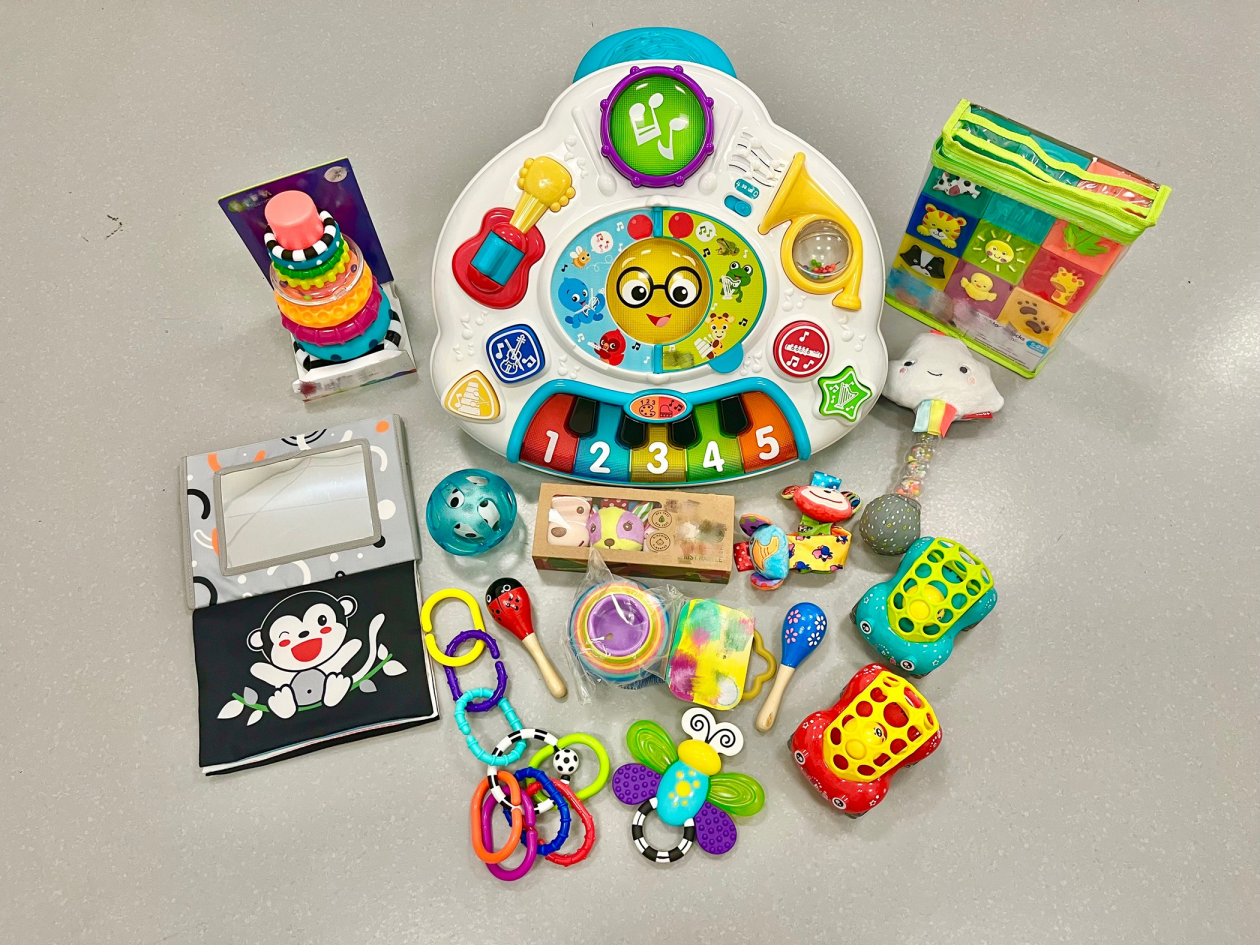
Example of toy kits provided to families as part of this study.

To support the principle of environmental enrichment, families in the intervention group will be provided with developmentally appropriate toy kits at three different time-points during participation. Toy kits include easily available items, such as a mirror, a high-contrast ball, rings, a book, a sound-making toy, and an activity table (Fig 2).

### 2.5 Intervention therapist training

#### Initial training

All intervention therapists are licensed physical therapists with pediatric experience. Prior to the study launch, intervention therapists attended a 2-day in-person training to review the study framework, intervention details, and data management. Additional individual review sessions were completed based on the setting of intervention implementation (NICU or home). Therapists joining the study after the initial staff training completed in-person or virtual training sessions to discuss the study framework and intervention details, review case scenarios, practice data management, and observe intervention sessions. Intervention materials, including caregiver resources and a therapist manual, are provided during training. Prior to leading intervention sessions, all therapists are required to pass a final evaluation, demonstrating an understanding of the study principles and guidelines.

### 2.6 Intervention fidelity monitoring

#### Recording and review of intervention sessions

We plan to video record 1–2 intervention sessions during the infant’s NICU stay. After NICU discharge, intervention sessions will be recorded monthly. The recordings will be reviewed by a data monitoring team, who will evaluate the integration and documentation of key principles during the session and complete a fidelity report. This will include completing the Pediatric Rehabilitation Instrument Measuring Engagement–Observation version (PRIME-O) [77] which is a 10-item instrument that uses a 0–4 scale to evaluate the engagement of the caregiver, the therapist, and the interactions between the two during therapy sessions. Additional training and evaluation will be completed if the therapist scores below 80% on this review. Once reports are reviewed and any issues addressed, recordings will be deleted to protect participant privacy.

#### Team meetings

Intervention therapists will attend 1–2 meetings per month, facilitated by a clinical lead, to review study key principles, discuss participant goals, problem-solve through case scenarios, and review themes arising from fidelity monitoring recordings.

#### Caregiver questionnaire

At each recorded session, caregivers will be asked to complete the Pediatric Rehabilitation Intervention Measure of Engagement–Parent version (PRIME-P), an 11-item self-report measure that asks caregivers about their level of engagement during the session and how the session affected their overall feelings about the intervention [78].

#### REDCap data review

Intervention dose, activity playbook resources assigned, level of caregiver engagement as evaluated by the Pediatric Rehabilitation Intervention Measure of Engagement - Service Provider version (PRIME-SP), and a therapist self-report on how well key principles were integrated into the session will be evaluated to determine intervention fidelity.

### 2.7 Standard-of-care therapy

All infants enrolled in the study will have access to recommended standard-of-care services. These services may include therapy intervention while in the NICU and/or referral to Early Intervention at hospital discharge for those with qualifying medical diagnoses or identified delays, as well as other outpatient therapy services as recommended by the infant’s medical and therapy teams. To differentiate between the study intervention and standard-of-care therapy, we will employ a variety of strategies. To evaluate standard-of-care services received at our enrollment sites, we will complete an electronic medical record review. When infants are at home, we will ask families to complete a monthly update survey describing their standard-of-care services (including physical, occupational, and developmental therapies). We may ask their standard-of-care therapists to complete a questionnaire describing their clinical approach and therapy goals; we may also ask to video those sessions. Videos and questionnaires will be reviewed by the study team to identify themes (such as strengthening, balance, or righting reactions).

### 2.8 Focus group

We will convene caregiver focus groups (in-person or virtual) for a subset of families in the intervention group, based on their interest in participation. We will send out a questionnaire to each participating family before the focus group that will ask for their feedback on the acceptability and practicality of the intervention, study procedures, and data collection sessions; integration and adaptability, implementation, and effectiveness of the study [79, 80]. The focus group will discuss the themes of resource utility, strength-based approach, caregiver engagement, the feasibility of dosage and time commitment, and general feedback on participating in the study.

### 2.9. Assessments and outcome measures

#### 2.9.1 Primary and secondary outcome measures

The timeline for conducting outcome measures and neurological assessments is shown in Fig 1. The results of any clinical assessments completed in clinic will be shared with families. Families may also request the results of assessments completed by study therapists. Video and sensor data will not be shared with participants.

*Bayley scales of infant and toddler development*, *4th edition*. The Bayley-4 is a widely administered, norm-referenced tool used to identify developmental delays in children aged 16 days to 42 months of age. It examines five domains including cognition, language, motor, social-emotion, and adaptive behavior [81]. Since the intervention is intended to target motor development, the primary outcome will be the Bayley-4 motor score, which evaluates both gross and fine motor function. Due to the interplay between motor and cognitive development, we will also evaluate the Bayley-4 cognitive score as a secondary outcome measure.

#### 2.9.2 Exploratory outcome measures

*Wearable sensor data and video data*. At every assessment timepoint, infants will perform different age-appropriate tasks as their movements are recorded by wearable sensors, a cell phone camera, and a depth camera. The wireless, low profile, lightweight wearable sensors (3.2 x 2.1 x 0.3 cm, 2.6 g) collect tri-axial acceleration data and angular velocity data at 200 Hz [82]. Data are recorded wirelessly with Bluetooth technology. The depth camera records videos at a resolution of 640*480 pixels, 30 frames per second in addition to depth information.

**Fig 3:**
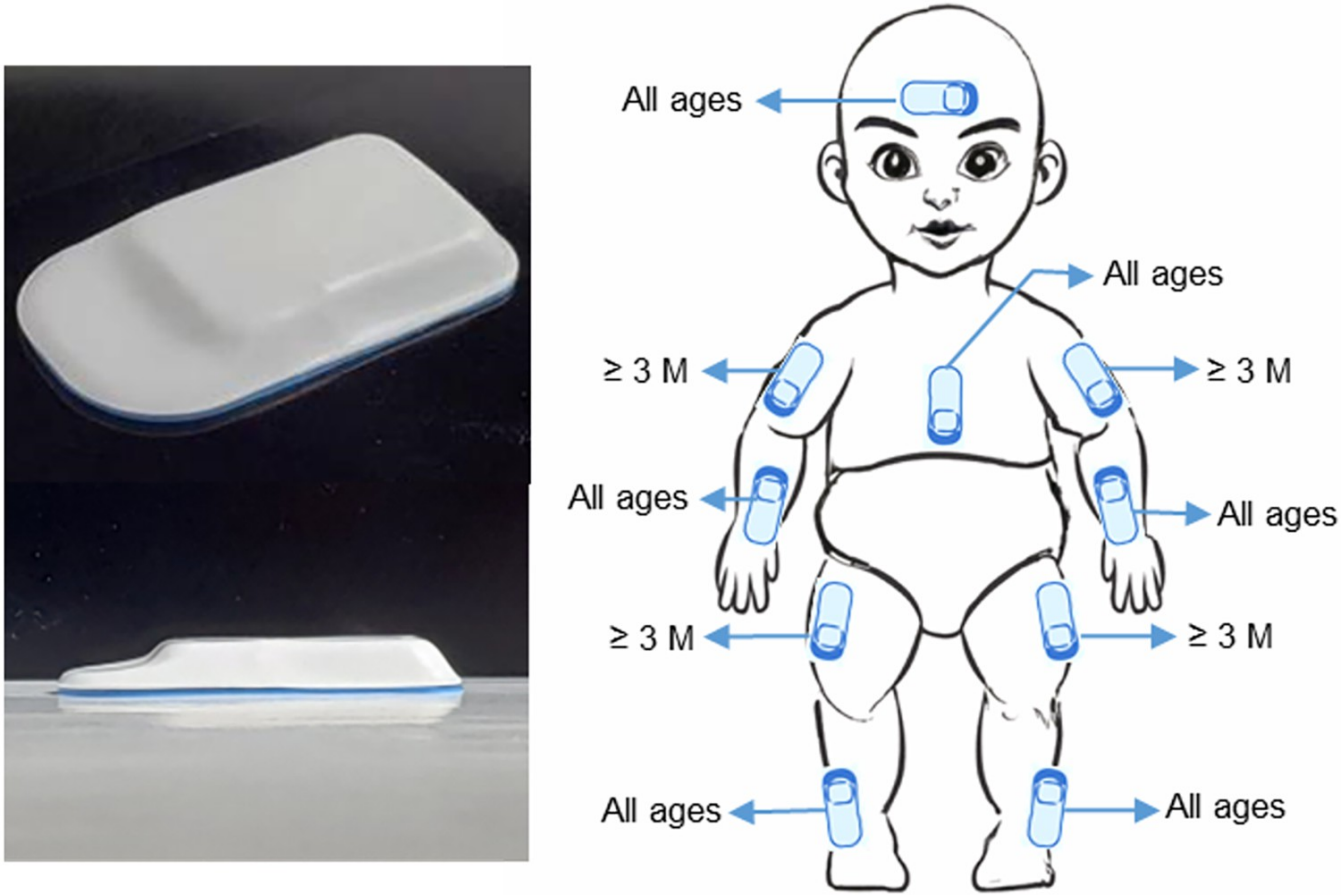
Sensor positions. Left: Lightweight wearable sensor. Right: Sensor placement locations. (3M: 3 months).

Either before, during, or after motor assessments, six to ten sensors will be placed on the limbs, head, and upper torso at standardized locations (Fig 3). Sensors will be held in place either via self-adherent wraps or adhered to the skin using hydrogel; the head sensor is placed under a hat. When visits take place in the NICU, a trained member of the NICU staff will apply sensors. When visits take place in the infant’s home or in the clinic, a study team member will apply sensors. For infants less than 3 months of age, we will place a sensor on each of their lower arms and legs and on their head and chest (for a total of six sensors). For infants at or above 3 months of age, we will place sensors on each of their upper and lower limbs and one on their head and chest (for a total of 10 sensors). Sensor data are collected during various positions and tasks, as shown in Table 4. In the NICU, a skin assessment will also be completed before and after sensor placement to monitor for skin irritation.

**Table 4:** Sensor data collection tasks.

Task	Duration	Location
Supine	Up to 5 minutes	NICU + Home
Prone	Two minutes	NICU + Home
Head control	Up to 30 seconds	NICU + Home
Pull to sit	Two trials	Home
Supported sitting	One minute each of front and side view	Home
Unsupported sitting	Up to 30 seconds	Home
Supported standing	One minute each of front and side view	Home
Ventral suspension	One minute	Home
Reaching	Up to 2 minutes	Home

As such, the sensors may capture the complex motor patterns that evolve during the first one-two years of life in a variety of positions. Three-dimensional, whole-body movement data from the sensors will be compared between the intervention and standard-of-care groups. Video data recorded during sensor data collection will also be used to develop algorithms for automated markerless pose estimation, which will further support comprehensive motion analyses early diagnosis, and outcome prediction [82].

*Test of infant motor performance (TIMP)*. The TIMP is an outcome measure used to evaluate the early effects of the intervention on motor performance. It is composed of 42 items, either observed or elicited, that assess posture and selective motor control in infants between 34-weeks and 4 months corrected age. Observed items are scored yes (1 point) or no (0 points); elicited items are scored from 0–3, 0–4, 0–5, or 0–6, with higher scores indicating better performance on the item. The maximum score is 142. A Z-score is calculated based on the infant’s age at the time of assessment and their total score [83, 84].

*Pediatric quality of life inventory (PEDS-QL) family impact module*. The PEDS-QL Family Impact module is a self-report instrument designed to measure the effect of pediatric chronic health conditions on caregivers and the family. It evaluates caregiver self-reported physical, emotional, social, and cognitive functioning; communication; worry; daily activities; and family relationships [85, 86]. The instrument is scored using a 5-point response scale (0 = never a problem; 4 = always a problem). Items are reverse-scored and linearly transformed to a 0–100 scale, so that higher scores indicate better functioning (i.e., less negative impact on the family). A total score, a parent health-related quality of life summary score, and a family functioning summary score are calculated [87].

This instrument will be used to evaluate the impact of an intervention focused on a strengths-based approach and caregiver engagement on caregiver well-being and quality of life.

*Alberta infant motor scale (AIMS)*. The AIMS is a norm-referenced, standardized, 58-item assessment evaluating gross motor function. This observational assessment is completed in four positions: prone (21 items), sitting (12 items), supine (9 items), and standing (16 items). For each subscale, items are scored as observed or not observed. Percentile ranks are calculated from the total raw score based on the infant’s corrected age, as needed. Cut-off scores may be utilized to identify if an infant is risk for motor delay [88].

*Neurological*, *sensory*, *motor*, *developmental assessment (NSMDA)*. The NSMDA is a criterion-referenced assessment that evaluates motor function and neurodevelopment. It is composed of five domains—neurological, postural, sensory, fine motor, and gross motor [89, 90]. It can be used in infants between the ages of 1-month and 6-years of age. Within each defined domain, a total functional grade is assigned. The sum of the functional grades provides a classification of motor disability defined as normal, minimal deviation, mild deviation, moderate deviation, severe deviation, or profound deviation.

*Pediatric evaluation of disability inventory (PEDI-CAT)–mobility subsection*. The PEDI-CAT is a computer-administered, caregiver-rated questionnaire that is used in children from 1-year to 21-years of age. Caregivers will evaluate the infant’s performance on a typical day by completing the mobility subsection. Evaluated items include rolling over and sitting unsupported, in addition to more advanced skills. Normative and scaled scores are included in the summary report [91]. Higher scores indicate better performance.

*Warner initial developmental evaluation of adaptive and functional skills (WIDEA)*. The WIDEA is a 50-item caregiver-rated questionnaire assessing a child’s adaptive skills in an everyday context. It can be used in infants from 0–36 months of age. It comprises 4 subsections including mobility (9 items), communication (13 items), social cognition (11 items), and self-care (17 items). The total score ranges from 50–200, with higher scores indicating better performance [92].

#### 2.9.3 Assessments to monitor infant neurologic status and covariates of interest

*General movement assessment (GMA)*. The GMA evaluates the integrity of the nervous system in young infants and can identify infants at risk for neuromotor delay [93]. From birth until 6 weeks post-term age, infants demonstrate motor patterns termed “writhing movements”, which are variable sequences of neck, arm, trunk, and leg movements that vary in their intensity, speed, and range of motion. From 6–8 weeks post-term age, writhing movements gradually disappear and “fidgety movements” emerge. Fidgety movements are minute movements that take place in all directions in the neck, trunk, and limbs. They are present until 20 weeks post-term age. Cramped-synchronous general movements and the absence of fidgety movements are significant predictors of cerebral palsy [94, 95].

*Hammersmith infant neurological examination (HINE)*. The HINE is a commonly used tool for infants between 2 months and 24 months of age [96]. It assesses different components of a neurological examination including cranial nerves, posture, movement, tone, and reflexes. Each Item on the assessment is scored separately with a minimum value of 0 and a maximum value of 3. Individual scores are summated to achieve a global optimality score (range 0 to 78). Global scores are rated as optimal if they are equal to or above 67 at 3 months, 70 at 6 months, and above 73 from 9–12 months. Scores are rated as atypical if they are equal to or below 57 at 3 months, 60 at 6 months, 63 at 9–11 months, or 66 at 12 months [97]. In addition, asymmetry scores may also be calculated and used to identify infants with hemiplegia [98].

*Electronic medical record review*. Study teams will complete a comprehensive EMR review process, which includes capturing neurophysiological imaging results and hospital course during the infant’s NICU stay.

*Covariates of interest*. Each infant’s risk of neuromotor delay (determined at baseline), responses to a social influencers of health questionnaire (SIHQ) completed at 3 months, as part of standard-of-care, and amount and type of standard-of-care services received will be evaluated as covariates to determine the effect of these variables, if any, on the primary and secondary outcomes.

### 2.10 Assessment therapist training

Assessment therapists responsible for completing clinical assessments will participate in inter-rater reliability training. This will include administering and scoring an in-person assessment as well as rating video-recorded assessments and comparing their scores to an expert rater. Reliability will be assessed at the start of the study and at annual intervals for the study duration to ensure consistent administration and scoring of all clinical assessments throughout the study.

### 2.11 Data collection and management

Clinical assessments and questionnaires will be completed in the NICU, the infant’s home, and within the early childhood clinic at the infant’s recruitment site. Clinic procedures will be modified where necessary to maintain the blinding of assessment therapists. Assessment sessions may be completed over several visits based on infant tolerance. Video data collected as part of the research study will be stored on a secured network. Study data will be entered into and managed through a REDCap electronic data capture tool [75, 76] hosted by the Shirley Ryan AbilityLab. REDCap (Research Electronic Data Capture) is a secure, web-based software platform designed to support data capture for research studies, providing (1) an intuitive interface for validated data capture; (2) audit trails for tracking data manipulation and export procedures; (3) automated export procedures for seamless data downloads to common statistical packages; and (4) procedures for data integration and interoperability with external sources. We will conduct regular audits to ensure data quality. These audits include monitoring for missing or out-of-range values and the accuracy of calculated fields.

*adverse event monitoring*. Study team members will monitor for adverse events during all participant interactions. Any adverse events that occur during an intervention or assessment session will be documented, reviewed by principal investigators, and reported to the IRB, when necessary.

*Participant withdrawal/discharge*. In the event of a significant medical status change in an infant that would impact study participation, or based on the family’s ability to adhere to study requirements, study neonatologists, and principal investigators will determine their continued involvement in the study. All standard-of-care therapy referrals will be managed by the infant’s primary care team. For infants enrolled in the intervention, study therapists will provide caregivers with a care summary at the intervention endpoint.

## 3. Statistical analysis

### Sample size calculation

The primary outcome measure, the motor subscale score of the Bayley-4 at 12 months, is a standardized score with a range of 45–155, a mean of 100 and a standard deviation (SD) of 15 but high risk children show more variable SD [99–101] and in a high risk clinic population from one study site, the SD for motor scores was 17.5 (unpublished data). For this study, we consider Bayley motor scores that are 8 points (~0.5 SD) higher in the intervention group compared to the control group at 12 months corrected age a clinically significant difference. For statistical power calculations, we used an SD of 17.5 to reflect the potential variability in Bayley-4 motor scores of infants enrolled in the trial. Given these pilot data sample size of 154 infants completing the trial (77 in each group) provides 80% power to detect an 8-point difference in the Bayley-4 motor scores at 12 months. To allow a 20% dropout rate, we will recruit up to 192 infants (96 in each group). Power calculations were completed using PASS 2020 software.

### Analysis

The primary analysis will be a comparison of the 12-month Bayley-4 motor scores between the two study groups. Intent-to-treat (analyzing results from all randomized participants regardless of what treatment they received) and per protocol analyses (v02-21-23) will be conducted; for the latter, evaluable data for the intervention group is defined as infants who complete at least 50% of intervention sessions. We will choose appropriate analysis methods (such as mixed effects model, etc.) based on the distribution of data, including covariates such as gestational age, risk of neuromotor delay, recruitment site, and sex. As Bayley 4 is not specifically designed to measure movement asymmetry, we may employ a composite score comprising multiple above-mentioned standard outcome measures that incorporate asymmetry parameters.

To address the second hypothesis regarding early differences in outcomes, we will summarize outcome measures at each time point by group. Linear mixed effects models with repeated measures may be used to compare score changes over time between groups and/or recruitment sites. Differences between groups at early time points will be estimated based on the fitted model by constructing appropriate contrasts and compared using post hoc tests. Separate models may be fitted for each outcome measure, as appropriate.

In addition, the dose-response relationship between the 12-month outcomes (response) and the number of home visits or total hours of caregiver-provided intervention during the 12-month period (dose) will be examined among patients randomized to the treatment group and/or recruitment site. Linear regression models may be adopted to fit with the 12-month outcome measure as the dependent variable, and the number of home visits/total hour**s** of caregiver-provided intervention as the predictor. Nonlinear terms may be included to model the potentially nonlinear association. These models may also be adjusted for important baseline prognostic covariates as for the primary outcome measure.

If there is substantial missing data or imbalanced data between the two groups, we may consider multiple imputation (which generates several plausible datasets with different estimated data for the missing values, and combines the results from the analysis of each dataset) and/or a matching technique (which selects a subset of the majority group, matched to participants in the minority group based on the risk of neuromotor delay and approximate intervention dosage as best as possible) to produce reliable and robust results.

## Discussion

This study will be one of the first clinical trials to investigate the efficacy of a one-year-long continuum of therapeutic intervention on the development of infants at risk of neuromotor delay. Our goal is to initiate the intervention in the NICU to take advantage of the high neuroplastic potential in early infancy [14–16], although this may be influenced by infant medical stability and length of stay. Some infants may begin the intervention after NICU discharge due to the timing of medical clearance, study consent process, and duration of NICU stay; however, we expect that most infants in the study will start the intervention at or before term age.

Our study combines several state-of-the-art evidence-based strategies, including caregiver engagement, active learning, environmental enrichment, and a strength-based approach. In addition, we will provide the intervention at a high dose, using both therapist- and caregiver-led components. Coaching and empowering caregivers to implement study-related activities throughout the day will provide as much opportunity as possible for the infant to practice motor skills.

Although the focus of this intervention is motor development as measured by Bayley-4, motor development and cognitive development are highly correlated, thus we may see effects in both the motor and cognitive domains [102–104]. In order to capture the potential effects of the intervention at earlier time points, we will also monitor developmental outcomes using additional clinical assessments, wearable sensors, and video technologies at several time points during the intervention. We will offer families the option of follow-up assessments at 18 and 24 months of age after the one-year-long intervention. The study will thus generate a wealth of data on neurodevelopment in a population of infants with diverse diagnoses and risk factors, from birth through their first one to two years of life.

In contrast to other intervention studies that focus on specific populations of high-risk infants [30, 43, 47, 58, 59], we will recruit infants with different risk levels. Although many infants at mild risk of neuromotor delay will develop typically [105], which may mask the effectiveness of the intervention, some infants at apparent mild risk may subsequently develop atypically. This transdiagnostic approach may allow us to identify which infants will benefit from early therapeutic intervention. The study population will also reflect the general population of infants admitted to the NICU, so that the results may be more broadly applicable.

To define standard-of-care therapy for infants in both study groups, we will use both questionnaires and video recordings of therapy sessions, where possible. Although some non-study-affiliated therapists may not agree to provide information or be recorded [106], if possible, this information may provide valuable insights into intervention therapies that are currently available for infants in our study population and define differences between standard of care and intervention.

This is an ambitious study that poses many unique challenges. This study may impose a significant burden on participating families, both to accommodate twice-weekly intervention therapist visits and to provide the necessary dose of caregiver-provided activities over a one-year timeframe. In addition, we ask families to participate in multiple assessments within their homes or the clinic. We will attempt to mitigate this burden by scheduling appointments to accommodate caregivers’ schedules and needs and by providing appropriate reimbursement to participating families, including monthly payments for remaining in the study. We will also hold focus groups to understand barriers and facilitators to participation, which may enable us to make appropriate modifications to study procedures.

This large-scale RCT may provide high-quality evidence of the efficacy and/or effectiveness of the Project Corbett Ryan Intervention for infants with various risk levels of developmental disabilities. We will evaluate the efficacy of this year-long intervention on motor and cognitive development, explore the early effects of the intervention, and investigate the effect of dosage on the effectiveness of this early intervention. Other secondary analyses will probe changes in motor development captured using wearable sensors and markerless pose estimation, and consider the influence of ecological factors. In the long term, the results could guide policymakers, professionals, and caregivers in the provision of early therapeutic intervention to improve infant developmental outcomes.

## Supporting information

S1 File (DOCX)
