# Supplementary material for: Protocol for a randomized controlled trial to evaluate a year-long (NICU-to-home) evidence-based, high dose physical therapy intervention in infants at risk of neuromotor delay
Source: PLoS One. 2023 Sep 19;18(9):e0291408. doi: 10.1371/journal.pone.0291408 (PMC10508609; doi:10.1371/journal.pone.0291408)
Supplement: S1 File — (DOCX) [file pone.0291408.s001.docx]

PROTOCOL TITLE: ***Early Detection and Therapeutic Improvement of Motor Delay in High Risk Infants: A Randomized, Controlled Trial***

# PRINCIPAL INVESTIGATOR:

*Name:* Raye-Ann O. deRegnier, MD

*Institution:* Northwestern University/Lurie Children’s/Prentice Women’s Hospital

*Department:* Pediatrics (Neonatology)

*Telephone Number:* 312-503-9807

*Email Address:* r-deregnier@northwestern.edu

# Other Site Principal INVESTIGATORS:

*Name:* Arun Jayaraman, PT, PhD

*Institution*: Shirley Ryan AbilityLab

*Telephone Number:* 312-238-6875

*Email Address:* ajayaraman@sralab.org

*Name:* John Rogers, PhD

*Institutions:* Northwestern University

*Department:* Materials Science and Engineering

*Telephone number:* 847-491-5220

*Email address:* jrogers@northwestern.edu

*Name:* Nishant Srinivasan, MD

*Institution:* Children’s Hospital at the University of Illinois

*Department:* Pediatrics

*Telephone number:* 312-996-4185

*Email address:* srinis@uic.edu

# VERSION DATE: 08/19/2022

# Study Summary:

| Investigational Agent(s)  (Drugs or Devices) | N/A |
| --- | --- |
| IND / IDE / HDE # | N/A |
| Indicate  Special Population(s) | Children  Children who are wards of the state  Adults Unable to Consent  Cognitively Impaired Adults  Neonates of Uncertain Viability  Pregnant Women  Prisoners (or other detained/paroled individuals)  Students/Employees |
| Sample Size | 240 |
| Funding Source | Patrick G. & Shirley W. Ryan Foundation |
| Indicate the type of consent to be obtained | Written  Verbal/Waiver of Documentation of Informed Consent  Waiver of HIPAA Authorization  Waiver/Alteration of Consent Process |
| Site | Lead Site (For A Multiple Site Research Study)  Data Coordinating Center (DCC) |
| Research Related Radiation Exposure | Yes  No |
| DSMB / DMC / IDMC | Yes  No |

# Objectives:

Project Corbett is a randomized controlled trial to evaluate the efficacy of a one-year long, evidence- and clinical experience-based intervention to improve motor function and reduce the severity of motor delays in infants who have been admitted to a Neonatal Intensive Care Unit (NICU). We will also assess movement impairments and function using outcome measures at various time points up to 24-months corrected age.

# Study Aims:

*Pilot study*: Due to the large recruitment goal and length of the project, the study team/PIs will evaluate the first cohort of 6-10 participants to refine study procedures and study-related materials. If no major modifications are made to the protocol as a result of this evaluation, data from these participants will be included for analysis.

*Aim 1****:*** Evaluate the efficacy of an early, evidence-based, clinical experience–based therapeutic intervention (from the NICU to 12-months corrected age) on improving motor function and reducing severity of motor delays in infants at 12-months corrected age.

Primary Outcome Measure:

Bayley-4 Motor Score at 12-months corrected age

*We hypothesize that the intervention group will demonstrate an average 8-point difference (0.5 standard deviation) compared to the standard of care group. [an 8-point difference is considered a clinically meaningful difference]*

*Aim 2*: Evaluate the early effects (i.e., before 12 months) of a therapeutic intervention, provided from NICU to 12-months corrected age, on motor function and severity of motor delay.

*We hypothesize that a statistically significant higher percentage of infants in the intervention group will demonstrate improved motor function and reduced severity of motor delays, compared to the standard of care group—assessed using sensors,* *the NSMDA and TIMP—as early as 3-months corrected age.*

*Aim 3*: Evaluate whether an early intervention that focuses on caregiver engagement improves caregiver well-being.

*We hypothesize that an intervention that focuses on supporting and addressing the individual needs of the caregiver will improve caregiver well-being. We will evaluate these effects using the PedsQL (Family Impact Module).*

**Background**:

Infants with typical neuromotor development exhibit so-called *general movements* (GMs), which are initiated by central pattern generators in the spinal cord or brain stem and modulated by the developing supraspinal circuitry. GMs evolve naturally during development, based on ongoing development of the brain. In pre-term and term infants, GMs are characterized by whole-body movements that vary in sequence, amplitude, and speed. Until 36-38 weeks post-menstrual age (PMA) these movements involve many movements of the trunk. After 38 weeks, GMs have more of a *writhing* quality, in which movement of the trunk is less obvious and movements become slower and smaller in amplitude. Between approximately 9 and 17 weeks post term, GMs are characterized by small movements in all directions that vary moderately in speed and acceleration—called *fidgety* movements. Fidgety movements are most prominent between 11-16 weeks post-term and disappear around 5 months post-term as they are gradually replaced by goal-directed, intentional, antigravity movements such as reaching.

Because the evolution of GMs in typically developing infants represents ongoing phases of motor development, atypical motor patterns in infants (i.e., as evaluated by the General Movement Assessment [GMA] or the Hammersmith Infant Neurological Examination [HINE]) in infants may reflect impaired motor development due to discrete clinical conditions (e.g., cerebral palsy, genetic disorders, autism spectrum disorder, or minor neurological dysfunction [MND]) or overall neuromotor delay. These conditions are highly associated with known risk factors, such as pre-term birth or damage to specific areas of the brain, which interrupt or disrupt these developmental processes.

Early theories regarded motor development as an innate, natural process. However, recent theories suggest that motor development progresses through phases that are affected variously by experiences, genes, and epigenetics. Abundant variability in movement in the typically developing infant provides rich self-produced sensory and proprioceptive feedback that in turn facilitates motor development. In later stages, this feedback information can be increasingly used to coordinate self-initiated, goal directed motor behavior; the typically developing infant is able to select from a wide repertoire of possible movements to accomplish a desired task. The infant can learn from their own movements as well as by imitating movements of others due to neural mirror networks that are already present at birth. Goal-directed movements develop sequentially, corresponding to different phases of development; e.g., postural adjustments emerge after 3 months, and reaching activities develop between 6-15 months. During these adaptive phases, the infant can increasingly incorporate sensory feedback from the environment in order to fine-tune intended movements.

Because of the high plasticity of the infant brain, early intervention may be an important strategy to overcome or minimize impairments resulting from early brain damage. Because development relies on sensory feedback—obtained through active movement and interaction with the environment and other individuals—opportunities for self-directed movement (active learning), environmental enrichment, and caregiver engagement are key intervention components. In addition, for infants at risk of neurodevelopmental delay, empowering caregivers through positive communication, focusing on achievement rather than impairment, may improve caregiver wellbeing, thus facilitating improved interactions between parent and infant.

The proposed intervention is therefore based on five principles: active learning, caregiver engagement, environmental enrichment, strengths-based communication between the therapist and caregiver that focuses on the achievements of the individual infant, and dose. In addition, the intervention starts as early as possible (in the NICU) to capitalize on neuroplastic potential, comprises a high-dose of therapist- and parent-delivered activities, and continues until 12-months corrected age to enable continued functional progression throughout the first year of life.

# Study Endpoints:

*Primary Study Endpoints:*

- Completion of the 12-month assessment time-point for up to 240 infants, across all sites.
- Analysis of the Bayley-4 motor scores at the 12-month testing time-point for up to 240 infants, across all sites.

*Secondary Study Endpoints:*

- Evaluation of whether the intervention results in a reduction in the severity of motor delays, compared to the standard of care group, that can be detected as early as 3-months corrected age.
- Evaluation of the intervention’s impact on caregiver health-related quality of life.

# Inclusion and Exclusion Criteria

## Screening

Infants admitted to the NICU will be screened by study team members at each respective recruitment site starting at 33-34 weeks PMA. They will be identified as appropriate for the study based on the following inclusion/exclusion criteria. Screening will be completed with an electronic medical record review. Families will not be approached by study staff until the infant is medically stable and the family has been cleared by the NICU staff as ready to approach.

## Infants

*Inclusion criteria*

NICU admission and qualifies for Early Childhood Clinic (NICU high-risk follow up clinic) or Early Intervention due to:

- BW <1500 grams

**OR**

- Disorders of the central nervous system

—Brain injury (including but not limited to extra axial hemorrhage, any grade intraventricular or intraparenchymal hemorrhage, stroke, hypoxic ischemic encephalopathy (HIE), meningitis)

- - HIE includes mild, moderate, severe exam on modified Sarnat exam, both cooled and non-cooled
  - includes “*at* *risk for HIE”* with 10-minute Apgar <7 plus pH<7.15 or base deficit >/=12.

—Brain developmental abnormalities (hydrocephalus, microcephaly, cortical dysgenesis)

—Cramped synchronous movements at term PMA

**OR**

- Bronchopulmonary dysplasia (BPD) defined as need for respiratory support at 36 weeks postmenstrual age in an infant born at <32 weeks of gestation.

**AND**

- Medically stable **AND** able to start intervention between 34-48 weeks PMA.

*Exclusion criteria:*

(related to inability to complete intervention, sensor placement, or clinic assessments)

- open wounds, skin condition precluding sensor placement
- immune deficiencies requiring protective isolation
- limb reduction defects
- Followed primarily in another clinic (including but not limited to meningomyelocele and related conditions/trisomy 21)
- bleeding disorders or ongoing need for anticoagulation
- palliative or hospice care (for life limiting conditions including, but not limited to trisomy 18, 13)
- known visual impairment at the time of enrollment
- DCFS custody
- No English-speaking caregivers
- Any other condition that would preclude participation in the study, as determined by the PI
- Previously enrolled in competing randomized trial with developmental outcome variables

Each child’s enrollment in the study will be approved by the child’s neonatologist.

Standard of care therapists

*Inclusion Criteria:*

- Therapist treating an infant enrolled in the study post-NICU, in either the standard of care group or the intervention group.

*Exclusion Criteria:*

- ​Not treating infant involved in the study

# Study Intervention(s)/ Investigational Agent(s):

***Summary:***

Project Corbett is a randomized controlled trial evaluating the efficacy of a one-year long, evidence- and clinical experience-based intervention to improve motor function and reduce the severity of motor delays in infants admitted to the Neonatal Intensive Care Unit (NICU). Eligible infants will be enrolled during their NICU stay. Randomization will be stratified based on the infant’s risk of neuromotor delay. Infants randomized to the intervention group will complete up to two visits per week and will be provided a set of worksheets and videos (the Activity Playbook) to promote functional progression. Parents will be asked to complete activities described in the Activity Playbook for at least 20 minutes per day 5 days per week, which will be progressed collaboratively between the therapist and caregiver. Infants randomized to the standard of care group will receive services as deemed appropriate by their supervising medical team. Both groups will be able to seek out and receive therapy or medical services outside of the study, without limitation; however, the study team will aim to monitor the frequency and dosage of any additional services received. All enrolled participants, regardless of group allocation, will complete assessments at defined time-points (shown in Table 1), up to 24-months corrected age.

***Intervention Group:***

The intervention will start in the NICU and continue for up to 12-months corrected age. It is divided into 3 blocks:

- **Block A** is defined as the period from enrollment until discharge from the NICU.
- **Block B** is defined as post-NICU until 3-months corrected age.
- **Block C** is defined as 3-months until 12-months corrected age.

All intervention sessions within each of the blocks will focus on providing a high dosage of intervention while incorporating a strengths-based approach that focuses on the principles of active learning, environmental enrichment, and caregiver engagement. Infants in the intervention group may also receive services as assigned by their managing care providers, including referral to Early Intervention or outpatient therapy services. Participants will not be prevented from receiving any service that they would otherwise be eligible for as a result of participation in this study. Study staff will monitor the frequency and type of additional services received using parent surveys. We will provide participants with contact information for study staff.

***Block A (NICU to discharge home):***

Participants will receive 1-2 visits per week in the NICU (with option for telemedicine for parent participation, if needed) based on infant status and tolerance. Primary goals of Block A will be to:

- Build caregiver competency in infant signal and stress cue management.
- Maximize and support caregiver confidence in infant interactions.
- Introduce variable positions to promote caregiver engagement and infant tolerance to movement.
- Promote active learning in different functional positions.

Intervention therapists will provide caregivers with written and video resources (the Activity Playbook), based on the infant’s abilities and tolerance, to promote functional progression. Intervention therapists may also provide supplementary materials related to infant state regulation, additional activities, and/or caregiver bonding, as necessary.

Activity recommendations will address the goals outlined above. Activities will be progressed appropriately by the intervention therapist and caregiver(s), based on collaboratively identified goals, caregiver needs, and the infant’s functional progression.

Caregivers will be asked to complete the Activity Playbook assigned by the intervention therapists at a target dosage of at least 5 days/week for at least 20 minutes per day. Caregivers will be asked to track the amount of time they spent on the activities.

Study staff will monitor caregiver engagement and intervention therapists will assist with any barriers to implementation of the Activity Playbook. Intervention therapists will communicate with the study medical team if they become aware of any additional needs that fall out of the scope of the study.

***Block B (post-NICU to 3-months corrected age) and Block C (3-months to 12-months corrected age):***

Participants will be provided with two in-person intervention sessions per week within their homes (in-clinic or telemedicine options may be available, if necessary).

Primary goals of **Block B** will be to:

- Build caregiver competency in infant signal and stress cue management.
- Maximize and support caregiver confidence during infant interactions.
- Introduce variable positions to promote caregiver engagement and infant tolerance to movement.
- Promote active learning in different functional positions.
- Collaborate with caregivers to facilitate integration of Activity Playbook recommendations throughout their day.

Primary goals of **Block C** include:

- Promote active learning in varied positions while progressing goal-directed behaviors.
- Collaborate with caregivers to maximize competency and consistency in completing Activity Playbook recommendations at recommended dosage.

Throughout the intervention session, intervention therapists will work with caregivers to progress infant motor development and facilitate goal-directed behaviors.

Intervention therapists will promote active participation of caregivers, while focusing on integration of the study principles.

Caregivers will be asked to complete Activity Playbook recommendations assigned by the intervention therapists at a target dosage of at least 5 days/week for at least 20 minutes per day. The therapist and caregivers will collaboratively progress the target dosage.

Intervention sessions and Activity Playbook recommendations will be modified appropriately by the intervention therapist, based on collaboratively identified goals, caregiver needs, and functional progression of the infant.

Study staff will monitor caregiver engagement and intervention therapists will assist with any barriers to implementation.

As a smartphone or other device (e.g., laptop, tablet) will be required to access videos and some study surveys, we will provide a smartphone and data plan to caregivers in the Intervention group, if necessary, until they complete the study (approximately 12-months corrected age).

Sensor data may be acquired during intervention sessions to evaluate infant movement. These sensors are described in the assessment section below.

***Assessment Timeline:*** Assessments will be completed at study enrollment and thereafter at monthly intervals up to 3 months corrected age, then at 3-months corrected age, 6-months corrected age, 9-months corrected age, 12-months corrected age, 18-months corrected age (optional), and 24-months corrected age (optional). The number of assessments prior to 3 months corrected age will vary by preterm status and age of enrollment. Optional assessments will be completed if (i) the study is still ongoing and (ii) the caregiver agrees to do so. If infants are unable to complete any required assessment sessions, an attempt to re-schedule will be made. If infants are unable to complete a testing session, their participation in the study will not be affected; however, the study team will appropriately document the missing data.

***Additional monitoring:*** Intervention therapists and study team members will monitor infant response to intervention sessions, caregiver engagement (caregiver- and therapist-report) compliance to key principles, percentage of intervention sessions completed, the dosage of Activity Playbook activities achieved (caregiver report), any adverse events/changes in medical status, additional services received outside of study participation, general medical and health updates, and additional information if relevant for study purposes. Surveys or medical chart reviews may be utilized to capture the above information throughout study participation.

***Standard of Care Group:***

Participants will receive services as assigned by their clinical team. Families will receive information on early intervention services, including speech therapy, occupational therapy, and/or physical therapy. The child will be referred by the clinical team, if eligible, and the family is interested in these services. Participants will not be prevented from receiving any service that they would otherwise be eligible for as a result of participation in this study. Study staff will monitor the frequency and type of additional services received.

For all participants, we will monitor access to recommended services and follow up if families report having difficulty accessing desired services and would like assistance. We will provide all participants with contact information for study staff.

*Assessment Timelin****e:*** Assessments will be completed at study enrollment and thereafter at monthly intervals up to 3 months corrected age, then at 3-months corrected age, 6-months corrected age, 9-months corrected age, 12-months corrected age, 18-months corrected age (optional), and 24-months corrected age (optional). The number of assessments prior to 3 months corrected age will vary by preterm status and age of enrollment. Optional assessments will be completed if (i) the study is still ongoing and (ii) the caregiver agrees to do so. If infants are unable to complete an assessment session, we will attempt to reschedule the appointment. If the infant does not complete an assessment session, their participation in the study will not be impacted; however, the study team will appropriately document the missing data.

*Additional monitoring:* Study team members will monitor for any adverse events/changes in infant medical status, general medical and health updates, and therapy services received outside of study participation. Additional information may be collected, if relevant for study purposes. Surveys or medical chart reviews may be utilized to capture the above information throughout study participation.

# Procedures InvolveD:

*Setting:* This study will have three primary recruitment sites including: Ann & Robert H. Lurie Children’s Hospital of Chicago (Lurie), Northwestern Medicine Prentice Women’s Hospital, (Prentice) and the Children’s Hospital at the University of Illinois (UIC). Shirley Ryan AbilityLab (SRAlab) will serve as the Data Coordinating Center. All sites will work collaboratively to manage logistics, recruitment, intervention development, intervention delivery, assessment, data analysis, and dissemination/publication of results. A data use agreement (DUA) will be in place for all actively enrolling sites to govern the transfer of research data between sites.

*Recruitment:* Study team members at each recruitment site will screen infants admitted to the NICU via an electronic medical record review. Staff will confirm participant eligibility criteria (see inclusion and exclusion criteria above) overall medical stability, and family readiness prior to approaching for study introduction and consent. Caregivers of eligible infants who are cleared to approach but not available during normal working hours may be initially contacted through written materials and/or telephone or email to (i) determine their general interest in study participation and (ii) to ask when they next plan to be in the NICU so that staff can arrange to approach the family in person. Recruitment monitoring will be completed either locally or in REDCap.

*Informed Consent*: Identified staff will provide a study overview, review participation requirements, and complete the informed consent with parents. It is anticipated that infants will be screened at approximately 33-34 weeks post-menstrual age (PMA). Infants may be consented up to approximately 48 weeks PMA. Participants will be considered enrolled in the study upon completion of the informed consent.

*Randomization:* Randomization will be completed after participants are consented. Participants will be randomized at their respective recruitment site. Neonatologists at Lurie, Prentice, and UIC will review infant medical history and stratify the infants based on their risk for neuromotor delay (mild or moderate-severe) using the following criteria, to ensure a balanced matching of impairments across study groups:

*Mild risk:*

- At risk for Hypoxic Ischemic Encephalopathy (HIE) (10-minute Apgar <7 plus pH<7.15 or base deficit >/=12)
- Clinical HIE diagnosis or meningitis with normal MRI and no seizures
- Subdural, subarachnoid, or subgaleal hemorrhage with otherwise normal MRI and no seizures
- Mild abnormalities on cranial ultrasound or MRI (including, but not limited to, non-cystic periventricular leukomalacia (PVL) or mild white matter injury)
- BW<1500 grams with grade 0, 1 or 2 intraventricular hemorrhage (IVH)
- No BPD or Jensen Grade 1 nasal cannula (NC) ≤ 2 liters per minute LPM) at 36 weeks

*Moderate-Severe risk:*

- Clinical HIE diagnosis or meningitis with abnormal MRI OR seizures
- Grade III/IV hemorrhage
- Cystic PVL
- Ventriculoperitoneal (VP) shunt
- Stroke
- Cramped synchronous movements
- BPD (respiratory support > 2 LPM NC at 36 weeks)
- Brain developmental abnormalities (hydrocephalus, microcephaly, cortical dysgenesis)
- Other surgical or medical condition likely to increase risk of neuromotor delay based on clinical judgement, at the discretion of the PI

*Intervention:*

*NICU:* The NICU intervention sessions (approximately 40 minutes to one hour) will be provided 1-2 times per week, based on caregiver needs and infant tolerance. Intervention sessions will be scheduled after completion of baseline assessment until discharge from the NICU. Intervention therapists may work with project coordinators, allied health professionals, and caregivers for appropriate timing of the intervention sessions. An attempt to reschedule any missed sessions will be made. At the beginning of each session, intervention therapists will review any changes in infant status/state with caregivers and/or allied health professionals. They will work collaboratively with caregivers to identify the infant’s current state of arousal, monitor for significant and consistent stress signals during the session, and modify activity selection based on infant tolerance. Staff will record session outcomes and complete appropriate forms in REDCap. For infants that may require additional support during intervention sessions (infants who require respiratory support >2 LPM nasal cannula and others based on bedside nurse recommendation) a research nurse will attend the intervention or assessment to monitor the infant.

*Home:* The Block B and C intervention sessions will be provided up to 2 times per week, until 12-months corrected age. Intervention sessions will be scheduled based on staffing and caregiver/infant schedules. Session attendance will be monitored and any missed sessions will be rescheduled if possible. Due to the length of the study, it is possible that infants may miss some intervention sessions. Intervention therapists will complete a subjective caregiver survey at the initiation of each session and will appropriately record session outcomes and update appropriate forms in REDCap.

*Activity Playbook*: In addition to the therapist-provided intervention sessions outlined above, caregivers will be provided with an Activity Playbook comprising video and written resources for activities. Completion of these activities will be tracked. If needed, the monitoring mode will be adjusted to fit the needs of the caregiver (e.g., myCap, paper log, phone call). As applicable, intervention therapists will assist in identifying strategies to address any challenges that caregivers may be facing in completing the activities or in achieving or progressing the target dose.

*Toy Kits*: We will provide developmentally appropriate toys to families in the intervention group periodically throughout the intervention phase, to enable families to provide environmental enrichment irrespective of family resources*.* Children in the standard of care group will receive developmentally appropriate toys at the end of their participation in the study, so that each child receives a similar number of toys over the course of the study.

*Mode of Intervention Sessions*: The primary method for intervention delivery will be in-person sessions at the participant’s home or NICU. However, based on the needs of the individual family or infant, sessions may be provided in-clinic (for those in Blocks B or C) or virtually (Blocks A, B, and C); this flexibility will maximize intervention dosage. If sessions are provided in the clinic, families will be reimbursed for parking expenses. Families will be screened for COVID-19 prior to each intervention session. If any member of the family is unwell or there is a known exposure to COVID-19, the session will be rescheduled or conducted via telemedicine.

*Documentation*: Primary data entry and storage will be completed through the SRAlab REDCap system. Paper surveys and forms will subsequently be added to REDCap by a member of the study team. Intervention forms/surveys may include, but not be limited to: a home environment checklist, demographics, subjective caregiver survey, visit summary, caregiver identified goals, PRIME-SP, PRIME-P (completed monthly by the caregiver(s)), adverse event log, documentation of dosage of caregiver-provided activities from Activity Book and documentation of any additional services received. (See supporting documents for details). Some intervention sessions and assessment sessions will be videoed for training purposes and fidelity checks. Adverse events will be documented and reported to the PI, IRB, and Sponsor, according to IRB guidelines.

*Minimization of risk*: This is a minimal risk study that involves provision of a physical therapy intervention and has no increased risk compared to other standard physical therapy interventions. Several strategies will also be implemented to minimize any potential risk to participants.

- Therapists will adhere to institutional vaccination policies and will self-screen before each intervention or assessment session.
- Intervention therapists and applicable study staff will complete a multi-day training on protocol guidelines and recommendations.
- Medical stability of infant and family readiness to approach will be evaluated prior to beginning the consent process.
- Collaborative communication with caregivers and/or allied health staff and/or medical chart reviews to monitor for any medical status changes or procedure updates within the NICU.
- Tolerance to therapy or assessment procedures will be monitored. If child experiences physiologic or behavioral responses or instability, the therapist will modify the procedures or end the session.
- Any unanticipated adverse event that results in discontinuation of the session will be documented.
- Infant response during intervention sessions will be monitored by the therapist and/or caregiver, and activities will be modified based on infant tolerance.
- Data will be maintained in a secure data capture system, with access limited to those identified as members of the study team.

*Audio/Video Recording*: Video and audio recordings will be used to evaluate the fidelity of intervention delivery and characterize standard of care therapy sessions.

- *NICU*: We will attempt to video record 1 session per week for approximately the first 6-10 infants enrolled in the study. Subsequently, we will attempt to video record 1-2 visits per infant during their NICU stay. Videos will be recorded via HIPAA-compliant platform Members of the Logistics & Management core will evaluate videos to monitor fidelity in intervention delivery, evaluate study feasibility, and assess the overall workload for video review. Videos will be stored on a secured, HIPAA-compliant server for review. Subsequently, videos will be stored on a HIPAA-compliant server. Only identified staff will have access to these videos.
- *Home*: We will attempt to video record the first 6-10 infants at least twice per month, to allow evaluation of intervention fidelity and protocol feasibility. Subsequently, we will record approximately 10-15% of sessions throughout the intervention phase of the study. Videos will be stored on a HIPAA-compliant server.
- *Standard of Care therapy:* Therapists providing standard of care services post-NICU will be invited to participate in recording sessions that will help the study team characterize the treatments. Data management procedures will be the same as those outlined above.

*Assessments*: All enrolled participants will follow the same assessment timeline (Table 1). Assessments will be completed at study enrollment and thereafter at monthly intervals up to 3 months corrected age, then at 3-months corrected age, 6-months corrected age, 9-months corrected age, 12-months corrected age, 18-months corrected age (optional), and 24-months corrected age (optional). The number of assessments prior to 3 months corrected age will vary by preterm status and age of enrollment. If infants are unable to complete a scheduled assessment session, efforts will be made to re-schedule. Flexibility in location (home vs. in-clinic) of assessment will be made available as necessary. Families will be screened for COVID-19 prior to each assessment session. If any member of the family is unwell or there is a known exposure to COVID-19, the session will be rescheduled or conducted via telemedicine where possible, following hospital guidelines. Assessments may be completed over several days depending on caregiver and infant needs. If infants are unable to complete a testing session, their participation in the study will not be impacted, however, the study team will appropriately document this issue. Assessment therapists will self-screen for COVID-19 symptoms or exposures before starting work each day, and will follow all clinic COVID-19 protocols. **Assessments in** **bold** in Table 1 are part of standard clinical care in the Early Childhood Clinic and would be done regardless of study participation (i.e., these are not study-specific procedures).

*Primary Outcome Measure:* Bayley-4 motor score at 12-months corrected age

*Secondary Assessments and/or Outcome Measures:* Sensors, PedsQL, Bayley-4 (cognitive)

*Exploratory Assessments and/or Outcome Measures:* GMA, NSMDA, TIMP, HINE, AIMs, PEDI-CAT, WIDEA-FS

*Assessments*:

| **Enrollment: Pre-term or Term Age** | **Monthly up to 3 months CA** | **3-months CA** | **6-months CA** | **9-months CA** | **12-months CA** | **18-months CA** | **24-months CA** |
| --- | --- | --- | --- | --- | --- | --- | --- |
|  |  |  | **Bayley-4^§^** |  | **Bayley-4^§^*** | **Bayley-4^§^** | Bayley-4**^§^** |
| **GMA**** | **GMA** | **GMA** |  |  |  |  |  |
|  |  |  | AIMS | AIMS | AIMS |  |  |
| Sensors | Sensors | Sensors | Sensors | Sensors | Sensors | Sensors | Sensors |
|  |  | NSMDA |  | NSMDA | NSMDA | NSMDA | NSMDA |
| TIMP | TIMP | TIMP |  |  |  |  |  |
|  |  | **HINE** | **HINE** | HINE | **HINE** |  |  |
| PedsQL (once during NICU) | | PedsQL | PedsQL | PedsQL | PedsQL | PedsQL | PedsQL |
|  |  | *****SIHQ** |  |  |  |  |  |
|  |  |  |  |  | PEDI-CAT | PEDI-CAT | PEDI-CAT |
|  |  |  |  |  | WIDEA-FS | WIDEA-FS | WIDEA-FS |

**^§^**Bayley-4 Motor, Cognitive, and Language modules only

*Bayley-4 motor score at 12-months corrected age is primary outcome measure

**GMA will not be scored when infant is 46-48 weeks PMA, but video and sensor data will be acquired

***SIHQ = Social Influencers of Health Questionnaire. Note that not all answers will be recorded as part of this study

**Note**: The number of assessments performed up to 3 months corrected age may vary based on age at enrollment

- **Bayley Scales of Infant and Toddler Development Fourth Edition (Bayley-4):** The Bayley Scales of Infant and Toddler Development Fourth Edition (Bayley-4) is a five-domain assessment tool used to determine developmental delays in children, from the age of 16 days until 42 months. The developmental domains covered in this assessment tool are cognitive, language, motor, social-emotional, and adaptive behavior. The cognitive, language, and motor domains are assessed by administering items to the child, whereas the social-emotional and adaptive behavior domains are assessed via caregiver-completed questionnaires: The assessment is in-person and takes approximately 15-40 min on average, with longer times for older infants. Infants may be video recorded during this assessment. This is a routine test in the Early Childhood Clinic.
- **The General Movements Assessment (GMA):** This assessment is designed to identify neurological issues that may lead to delay (i.e., cerebral palsy or developmental disabilities). Appropriate age range for assessment is from birth until 20 weeks CA. For the assessment, the infant, ideally in a calm alert state, is placed in a supine position and video-recorded from above. Generally, the video recording is for approximately 5 minutes, however, the infant may be recorded for up to 30 minutes to achieve an accurate representation of the infant’s movements. This assessment will be performed twice, once while the infant is wearing sensors and once without sensors. Individuals formally trained in the GMA will score the assessment. This is a routine test at term age in the NICU and at 3 months corrected age in the Early Childhood Clinic.
- **The Hammersmith Infant Neurological Exam (HINE)**: The HINE comprises 26-items, spanning five domains: cranial nerve function, posture, quality and quantity of movements, muscle tone, and reflexes and reactions. Each item is given a score between 0 and 3 based on a rubric, with a maximum possible summed score across all items of 78. Infants will be video recorded. This is a routine test at all study ages in the Early Childhood Clinic.
- **Neurological, Sensory, Motor, Developmental Assessment (NSMDA)**: The NSMDA measures neurodevelopment between 1-month and 6-years of age. It is composed of five domains: neurological, postural, sensory, fine motor, and gross motor. Each domain is provided a total functional grade of normal, minimal deviation, mild deviation, moderate deviation, severe deviation, or profound deviation. This assessment demonstrates predictive validity for long-term motor development. This test is often but not always part of the Early Childhood Clinic assessment.
- **Alberta Infant Motor Scale (AIMS):** The AIMS is a norm referenced, standardized, gross motor assessment. Infants are observed in four positions: prone, sitting, supine, and standing. For each subscale, items are scored as “observed” or “not observed”. The items in the observed range create a motor window. A total raw score is calculated and a percentile rank is given based on a sample of 2202 infants from Alberta, Canada. The AIMS has been reported to have reliability, concurrent and predictive validity. The duration of this assessment is approximately 10-20 minutes. Infants will be video recorded. This is not a typical Early Childhood Clinic test.
- **Test of Infant Motor Performance (TIMP):** The TIMP is a 42-item assessment of postural and selective motor control of functional performance in infants between 34 weeks and 4 months corrected age. Items are either observed (13) or elicited (29). Observed items (1-13) are scored yes (1 point) or no (0 points); elicited items (14-42) are scored from 0-3, 0-4, 0-5, or 0-6). Item scores are summed, and infants receive an age standard score, based on performance, of average, low average, below average, or far below average. The maximum possible score is 142. This is not typically done in the Early Childhood Clinic.

*Sensor Data*:

- Sensor data acquisition will take place at every assessment time point. Infants will wear wireless, lightweight sensors that record continuous, real-time movement information. Either before, during, or after motor assessments, up to 10 sensors will be applied by a study staff member on the limbs, head, and upper torso at standardized locations either via soft silicone bands, self-adherent wraps, or adhered to the skin. When the visits take place in the NICU, a trained member of the NICU staff will apply the sensors. When the visits take place in the infant’s home or in-clinic, a study team member will apply the sensors. A skin assessment will also be completed pre- and post- sensor utilization in the NICU. The infant’s movements will be recorded in various postures, as appropriate to the age/abilities of the infant, such as: supine, prone, pull-to-sit/sitting, standing, and ventral suspension. Manual support to the infant will be given as needed (e.g., supported sitting or supported standing). Sensor data will be recorded wirelessly with Bluetooth technology for no more than 60 minutes continuously while the infant is videotaped using two cameras–a traditional RGB camera and a stereovision camera that measures depth. A card with a subject ID may be placed next to the infant for labeling purposes. If the sensors would interfere with any therapeutic or monitoring equipment, they will be relocated if possible or removed for that visit, but video recordings will still be made. The sensor study is done solely for this project and it is not part of the Early Childhood Clinic routine.

### Surveys and Questionnaires:

- **PedsQL Family Impact Module** was designed to measure the impact of pediatric chronic health conditions on parents and the family. This 36-item module measures parent self-reported functioning in physical (6 items), emotional (5 items), social (4 items), and cognitive (5 items) domains, communication (3 items), and worry (5 items), as well as two scales measuring parent-reported family functioning: daily activities (3 items) and family relationships (5 items). The survey is scored using a 5-point response scale (0 = never a problem; 4 = always a problem). Items are reverse-scored and linearly transformed to a 0–100 scale (0 = 100, 1 = 75, 2 = 50, 3 = 25, 4 = 0), so that higher scores indicate better functioning (i.e., less negative impact). Scale Scores are computed as the sum of the items divided by the number of items answered, to account for missing data. The Total Scale Score is the sum of all 36 items divided by the number of items answered. The Parent HRQOL Summary Score (20 items) is computed as the sum of the items divided by the number of items answered in the Physical, Emotional, Social, and Cognitive Functioning Scales. The Family Functioning Summary Score (8 items) is computed as the sum of the items divided by the number of items answered in the Daily Activities and Family Relationships Scales. This is not a routine questionnaire in the Early Childhood Clinic and is being done for study purposes only.
- **PEDI-CAT (Mobility)** is a computer adaptive test (CAT) version of the Pediatric Evaluation of Disability Inventory (PEDI) that measures abilities in three functional domains: daily activities, mobility, and social/cognitive. The PEDI-CAT is designed for use in infants from birth (to 20 years of age) with a variety of physical or behavioral conditions. It is used to identify functional delay and to determine effects of an intervention. The PEDI-CAT mobility domain, which will be administered in this study, comprises 75 items in several content areas, including basic movement, standing and walking, steps and inclines, and running and playing. The survey is completed by the caregiver. The PEDI-CAT software utilizes Item Response Theory (IRT) statistical models to estimate a child’s abilities from a minimal number of the most relevant items or from a set number of items within each domain. This is not a routine questionnaire in the Early Childhood Clinic and is being done for study purposes only.
- **Warner Initial Developmental Evaluation of Adaptive and Functional Skills (WIDEA-FS):** The primary purpose of the WIDEA-FS is to assess a child’s adaptive skills in everyday contexts and it is completed by the caregiver. It evaluates four domains, including: mobility (9 items), communication (13 items), social cognition (11 items), and self-care (17 items). It has established construct validity and concurrent validity with the Bayley-III. Cut-off scores for both mobility and social cognition domains have been determined. This assessment is predictive of Bayley-III scores less than or equal to 85, when assessed at 10-18 months. The assessment takes approximately 10-15 minutes to complete. This is not a routine questionnaire in the Early Childhood Clinic and is being done for study purposes only.
- **Social Influencers of Health** is a demographics questionnaire in the Lurie EPIC electronic health record system that records information such as socioeconomic status, employment status, educational levels of the caregivers, etc., which may have a bearing on health outcomes. The Early Childhood Clinic asks about the Social Influencers of Health at the first visit to the clinic. We will not record responses to questions about physical or sexual abuse or presence of guns in the home for the study, as this information is not relevant to the study.

Other collected data during assessments will include:

- Videos of infants during assessment procedures (Table 1).
- Surveys to access participant updates, additional services received, family characteristics, including sociodemographic, structural, and home environmental factors as well as other salient behavioral dimensions of the caregiver(s) (e.g., social support, stress, mental health, wellbeing, family relationships, pregnancy course including expected due date). This includes the Social Influencers of Health completed during the 3-months corrected age time point. Other information as relevant to participation may also be included.
- Infant medical information: medical history, gestational/birth history, maternal health history, hospital course/complications, laboratory results, dates, related to the infant's condition, services received, and other information relevant to research via EHR documentation.
- Maternal medical information, including information on pregnancy course, complications, including Covid-19 and cytomegalovirus (CMV) exposures/infections, Covid-19 vaccination status and dates of vaccination, expected due date, and other information relevant to research via EHR.
- Skin assessment pre- and post- sensor use.
- Names and contact information including but not limited to: full names, home address and zip code, caregiver telephone number, caregiver email address via EHR documentation, confirmed by the caregiver.
- Information from therapists providing standard of care on the type and dose of therapy provided and the caregivers’ engagement and/or participation in therapy. Therapists may also be invited to participate in video recording sessions.
- Caregivers for the first 6-10 infants will be asked to participate in focus groups to obtain feedback on the caregiver experience, study materials, study protocols. This information will be used, as necessary to update study procedures and materials.
- Additional information may be collected as relevant to research.

*Assessment Session Structure and Duration:*

Assessment sessions will be completed within the NICU, the infant’s home, or at in-clinic visits once the child is discharged to home. If assessment sessions coincide with a standard clinic visit, we anticipate the physician appointment to take approximately take 20-30 minutes to complete. It is anticipated that study-related assessment sessions may require up to 90-120 minutes of additional time. Session duration may be modified based on infant needs and assessments may be performed over more than one day, if needed. For specific assessments performed at each time point, please refer to Table 1.

*Session Compensation*:

No compensation will be provided for participating in intervention sessions, but these approximately one-hour therapy sessions will be provided free of charge whether in the home, virtually, or in the hospital/clinic physical therapy department. If participants attend in-clinic visits and incur parking costs, we will reimburse them for the cost of parking. All participants (intervention group and standard of care group) will be compensated at a rate of $100 per assessment time-point. Participants may also be provided with a parking voucher. All payments will be provided by SRAlab via a ClinCard upon completion of the set of assessments corresponding to each time point (assessments may be completed over more than one session, if necessary). Infants in both groups, may be provided a small book or toy at the time of assessment visits.

# Data and Specimen Banking

The primary storage platform for study data will be the SRAlab REDCap (Research Electronic Data Capture) system. Any forms/paper documentation will also be maintained within study binders and/or uploaded into REDCap. REDCap is a secure web-based application for building and managing online databases for research studies. REDCap can be used to collect virtually any type of data and is specifically geared to support online or offline data capture for research studies and operations.

Sensor data will be stored and maintained by Dr. Arun Jayaraman’s group (at SRAlab) for analysis. Videos acquired during collection of sensor data will be maintained on a secured HIPAA-compliant AWS.

De-identified data or limited datasets will be shared with other investigators. Investigators from outside the study will be asked to sign an agreement of confidentiality in order to receive the de-identified data or limited datasets requested. Data use agreements will be in place between all actively enrolling, participating sites and SRAlab. The PI will be responsible for receipt and transmission of data. Data will be stored for at least 7 years. We plan to archive de-identified data in a data repository for future use by other researchers, after the study is completed.

No specimens will be collected as part of this study

# Sharing Results with Participants

Results of assessments completed during assessment sessions will be provided to families. during the clinic visit, as available. Some assessments require calculation of results, which will be done after the session and these results will be made available on request. Assessment results will also be provided to the child’s clinical care team if performed in the home, outside of the clinic. Information obtained from sensor data will not be provided to families.

The assessments completed during the course of this study may indicate a generalized diagnosis of motor delay or a specific diagnosis, for example, cerebral palsy. Given that we intend to perform the assessments in the Early Childhood Clinic, most families will be informed of this by the Early Childhood Clinic team. If the assessments are performed in the infant’s home due to a missed clinic visit, this information will be provided to the infant’s medical team, who may discuss these findings with the family and ensure the appropriate referrals are made.

# Study Timelines

Infants may be involved in the study for up to two years corrected age. The intervention group will receive therapy at a target dosage of 2 X per week until 12-months corrected age; both intervention and standard of care groups will participate in assessments until 12-months corrected age. Participants may also be asked to complete optional assessments at 18-month and 24-months corrected ages, if these time points occur while the overall study is still ongoing. These later time points are not required as part of the study, but may provide useful information on the longer-term effects of the intervention.

# Vulnerable Populations

The research involves infants from birth to 24-months corrected age who are at risk of motor or other developmental delay.

# Participant Population(s)

| Accrual Number: | Category/Group:  (Adults/Children Special/Vulnerable Populations) | Consented:  Maximum Number to be Consented | Enrolled:  Number to Complete the Study or Needed to Address the Research Question |
| --- | --- | --- | --- |
| Local: Lurie/Prentice  UIC | Infants | 180 | 123 |
|  | Infants | 60 | 31 |
| Total: |  | 240 | 154 |

# Recruitment Methods

The primary recruitment method is noted above under screening procedures. All participants will be identified during their NICU stay. Participants may be consented up to 48 weeks PMA.

# Compensation for Participation in Research Activities

For intervention visits, the cost of parking may be reimbursed for families who complete visits within a clinic setting. If cost of transportation is an issue for any family, reimbursement may be offered, on an individual basis. All participants (intervention group and standard of care group) will be compensated at a rate of $100 per assessment time-point. Participants may also be provided with a parking voucher. All payments will be provided by SRAlab via a ClinCard upon completion of the set of assessments corresponding to each time point (assessments may be completed over more than one session, if necessary).

In addition, payments will be made on a monthly basis to all participants who remain in the study, according to the following schedule: months 1-3: $25 per month; months 4-6: $50 per month; months 7-9: $75 per month; months 10-12: $100 per month.

If participants miss an assessment time point, they will not be reimbursed for the assessment visit. If they do not comply with other study requirements, such as completing surveys, their monthly payments will be stopped until their next scheduled study activity is completed. Monthly payments and assessment payments will resume after they complete the next scheduled study activity.

Focus group participants will be paid $100 per session completed. Parking may be reimbursed if applicable.

Participants will be informed that payments in excess of $600 in a calendar year are reportable to the IRS as taxable income

# Withdrawal of Participants

Participant families will be informed that their participation is strictly voluntary and that they can withdraw from any experiment at any time for any reason without consequence. Participants may be withdrawn from the research based on PI discretion regarding medical stability or compliance to study requirements. If a child is transferred from the NICU to the Pediatric Intensive Care Unit (PICU), they will be dropped from the study.

If subjects are withdrawn from the study after data has been collected, the data will be retained if it is deemed to be scientifically relevant by the researchers. Data that is not relevant will be discarded.

# Risks to Participants

This is a non-invasive, minimal risk study that does not involve collection of specimens. Potential risks and associated prevention or amelioration strategies are listed below.

Risk of harm due to intervention or assessment procedures

There is no significant increase in risks associated with participation in intervention sessions compared to standard physical therapy sessions. Some potential risks include:

For infants who are intubated, there is small risk of unplanned extubation. This will be minimized by the presence of the study nurse who will monitor the infants position and stability during the therapy sessions. For infants with respiratory or nutritional support (e.g., nasal cannula, NG, or G-tubes) there is a small risk of pulling the tube out of position or site irritation/bleeding during the intervention, standard of care therapy, or assessments. Care will be taken in handling of the infant at all times, and any indication of physiological changes that do not self-resolve or any additional concerns will be immediately reported to the study team and/or medical assistance will be requested, as appropriate. All study staff will be appropriately trained, and experienced at working with infants.

If a child has surgery, the intervention will be paused and surgical team clearance will be obtained, as appropriate before resuming study activities. If a child undergoes a procedure to remove a G-tube, study therapists will follow-up with the surgical team and modify the intervention or assessments based on their guidance. All surgical interventions will be documented.

Additional risks to participation include infant skin irritation, fatigue, or frustration. Intervention and assessment sessions will be completed according to infant tolerance. Changes in behavior state or stress cues will result in modification of the therapy session, as needed. Any loss of balance/falls with upright activities (e.g. stepping) will not be considered adverse events, as they are developmentally appropriate.

All assessments are standard infant outcome measures and as such have no known risks to the infant.

Risk of infant distress while wearing sensors

Risks associated with the use of the wearable sensor are minimal. Bluetooth technology is commonly used in hospitals, and it is compliant with FDA guidelines as well as FCC regulations. Though not expected, infants might experience discomfort or skin irritation from wearing the sensors. Based on infant response, the study team or caregiver(s) may request the sensor removal at any time. During the NICU phase, infants will be continuously monitored by telemetry and/or NICU staff. If an infant demonstrates significant distress, sensor use will stop and calming activities will be used to minimize infant distress. If the infant continues to express discomfort or distress, sensor data will not be collected at that study visit. The study team will monitor for signs of discomfort or skin irritation, as noted above, during study visits performed in the home or clinic.

Risk of skin irritation due to sensor placement

The Neonatal Skin Condition Scale (NSCS), an assessment used to objectively evaluate an infant’s skin integrity based on level of dryness, erythema, and breakdown, will be used as a safety measure to check for adverse effects due to sensor-skin contact on subjects in the NICU. A baseline NSCS score will be obtained by NICU/study staff before sensors are placed on the infant, as well as immediately after removal of the sensors and 30 minutes after removal of the sensors. Additional evaluation may be completed, as needed.

Risk of Caregiver discomfort when answering questions about their infant or themselves

Answering survey questions about their infant’s abilities or challenges, or the impact of these on the family and caregiver’s wellbeing may be uncomfortable. Caregivers will be advised that they do not have to answer any questions that they do not wish to answer.

### Risk of loss confidentiality

To mitigate this risk, caregivers and infants will be assigned and identified by a unique study ID. The key that links caregiver or infant names with study ID will be password protected on a secure server housed in SRAlab or at the infant’s recruitment site, and accessed only by authorized, IRB-approved study personnel. All electronically stored data, emails, or participant records will be double password protected. Any paper copy documents will be stored in locked cabinets at the respective sites; documents containing identifiable information will be stored separately from study data. All data will be analyzed and presented in aggregate form, with no identifying information attached that would reveal a participant’s identity.

Note: The only exception to participant confidentiality is that we are legally obligated to report evidence of child abuse or neglect. We will not ask caregivers about child abuse or neglect, but if caregivers tell us about child abuse or neglect we are required by law to report their name to state authorities. In addition, should caregivers report thoughts and plans of harming themselves or others, with imminent risk to their baby or someone else, we may take additional steps to ensure the safety of the caregiver, baby, and/or others. We will also ask caregivers if they would like to be with us when we contact the proper authorities.

# Potential Benefits to participants

We cannot guarantee any benefit to participation in the study or proposed intervention. However, infants in the intervention group may benefit from the high-dose, individualized therapy based on scientific evidence and clinical practice. Based on caregiver responses to the monthly parent subjective form, we will attempt to facilitate access recommended services outside of the study for all participants.

# Data Management and Confidentiality

***Data Analysis Plan***

### Power Analysis:

The motor subscale score of the Bayley-4 at 12-months corrected age is the primary outcome measure. It is a standardized score with a range of 45-155, a mean of 100, and a standard deviation (SD)=15. For this study, we will consider a significant effect of the intervention to be a Bayley motor score that is 8 points (~0.5 SD) higher in the intervention group than in the control group at 12-months corrected age. Assuming similar variability in this clinical trial with SD=15, a sample size of n=77 subjects per arm will provide 90.1% power with two-sided α=0.05 significance level to detect an 8-point difference in Bayley-4 motor scores at 12 months based on a two-sample equal-variance t-test. This would correspond, for example, to an improvement from the average 12-month Bayley-4 Motor score of 87.7 among patients (n = 77) recently assessed in our Early Childhood Clinic to an average score of 95.7. If we conservatively assume larger SD=17.5 due to possibly higher variability in Bayley-4 Motor scores in this high-risk population, we would still have sufficient 79.4% power to detect an 8-point difference between treatment arms. Assuming 30% attrition, up to 220 patients (110 per arm) will be required in order to obtain 154 patients evaluable for primary outcome. Assuming 20% attrition, up to 192 patients (96 per arm) will be required in order to obtain 154 patients evaluable for primary outcome. Power calculations were done using PASS 2020 software.

### Randomization:

Participants (including those in the pilot study) will be randomized to the two treatment arms using the method of permuted blocks. Randomization sequences will be generated by a statistician, and will be uploaded into the REDCap randomization module. Randomization will be stratified by study site and by risk of neuromotor delay (mild vs. moderate/severe). Siblings in multiple births will be assigned to the same group, and outcomes assessed only for the infant with the most severe risk. In order to ensure unbiased evaluations, assessments of motor function and other outcomes will be performed by different study team members than those administering treatment, and although this is not a blinded study, therapists conducting study evaluations will be blinded to treatment arm assignment whenever possible.

### Data analysis:

The primary analysis to compare 12-month Bayley-4 Motor scores between treatment arms, will be performed using a two-sample two-sided t-test assuming equal variance. Because the Bayley-4 scores are standardized, we expect them to follow the normal distribution. If the normality assumption is not satisfied, the Wilcoxon ranksum test will be used instead. In addition, a covariate adjusted estimate will be obtained using a linear regression model adjusted for known baseline prognostic factors, e.g. risk of neuromotor delay, gestational age at birth, and sex.

Baseline characteristics, study outcomes and treatment compliance rates will be summarized by treatment arm using basic descriptive statistics, e.g. mean, SD, median and inter-quartile range (IQR) for continuous variables, and frequencies and proportions for categorical variables.

To address questions posed in Aims 2 and 3 regarding early differences in outcomes, outcome measures will be summarized at each time point by group, and linear mixed effects models with repeated measures will be used to compare score changes over time between arms. In these models, the outcome measure, e.g., sensor data, PedsQL, GMA and others, will be the dependent variable, and predictors will include treatment arm and assessment time point as fixed effects and subject as the random effect. Within-subject correlation between repeated measurements will be accounted for by using an appropriate variance-covariance structure, e.g. autoregressive order 1 (AR(1)). Interactions between time and treatment may also be included as a fixed effect. Additional baseline prognostic covariates may also be included in the model as fixed effects to obtain covariate-adjusted estimates that may be more efficient. Differences between arms at early time points will be estimated based on the fitted model by constructing appropriate contrasts, and compared using post hoc tests. Separate models will be fitted for each outcome measure. Of note, this approach appropriately deals with missing data by including all evaluable observations across time points, rather than performing a complete case analysis. Analyses will be conducted using PROC MIXED in SAS statistical software. P-values will not be adjusted across these outcome measures because these are pre-specified analyses for outcome measures that address different aspects of the intervention.

In addition, the dose-response relationship between the number of home visits during the 12-month period (dose) and 12-month outcomes (response) will be examined among patients randomized to the treatment arm. Data will be graphically examined using scatter plots with an overlaid lowess smoother, and linear regression models will be fitted with the 12-month outcome measure as the dependent variable, and number of visits as the predictor. Nonlinear terms may be included for the number of visits (x-variable) to model the potentially nonlinear association. These models may also be adjusted for important baseline prognostic covariates as for the primary outcome measure.

### Interim Analysis

Interim analysis of the study is planned according to the alpha spending rule. The p-values are constructed to maintain the overall study power of 80% with alpha level of 0.05, two-sided. The interim analysis is planned to be conducted at end of approximately 12 -18 months, depending on enrollment. This analysis will focus on controlling type I error (alpha). We will use group sequential design with Pocock’s approach. If we repeat the significance tests at a fixed level, it will increase the probability of obtaining a significant result under the null hypothesis. In other words, it will increase the error rate. Thus, the goal is to conduct these repeated significance tests while maintaining corresponding error rate. Pocock applied the repeated significance tests to group sequential trials with equally spaced information levels and derives a constant critical value on the standardized normal *Z* scale across different analytic stages that maintains the Type I error probability level. For example, with a significance level 0.05 in a two-sided test, the derived critical value at one stage is 2.413 on the standardized normal *Z* scale, which is larger than the fixed-sample critical value 1.96. The corresponding nominal *p*-value is 0.0158, which is smaller than the fixed-sample *p*-value 0.025. Sequential design will be carried out in SAS using PROC SEQDESIGN.

### Data Management:

All personal information and study documentation that can identify participants will be kept secure to protect their privacy. Data collected during the study and shared with others will be de-identified and reference participants only by an alphanumeric code. The “master list” linking personal information to the alphanumeric code will not be shared outside of the study team.

Data will be collected and stored in compliance with HIPAA requirements. All collected data will be de-identified, stored in locked cabinets at the SRAlab with access limited to research staff, stored on password-protected computers (electronic folders will be private with limited access as determined by the PI) or stored in REDCap.

Access to REDCap data will be limited to study staff members who need access to complete study requirements, including data analysis. Data Use Agreements between actively enrolling study sites will allow SRAlab staff access to de-identified data from Lurie, Prentice, and UIC. Videos used as part of the Activity Playbook will be stored on Vimeo Premier, which is password protected. Videos of study participants recorded for assessment or fidelity checking purposes will be stored on a secure, HIPAA-compliant web server. These videos will not be shared unless the caregiver provides permission as part of the consent process.

Intervention session videos for fidelity will be destroyed after review and evaluation. Assessment session videos for fidelity will be kept until the end of the study and then destroyed.

Videos taken during sensor assessments and electronic data from sensors will be stored on a SRAlab secure research server and/or HIPPA-compliant Amazon Web Services platform.

Parent-reported data and forms completed by therapists will be entered into REDCap, a web-based data collection tool, which is supported by SRAlab. Neurodevelopmental assessment scores and comments, as well as but not limited to date of birth, expected delivery date, demographic information, and presence of risk factors, will also be stored in REDCap.

De-identified data will be stored indefinitely.

Data Quality Control:

Data collection will be reviewed by the study PIs and by the data safety monitoring board on a quarterly basis. Quality will be assessed based on completion of required study/assessment visits, completion of all necessary forms and their upload into REDCap, and collection of sensor data, if required. Data quality will be monitored through the use of data error/missing data reports through REDCap as well as specially developed quality control programs in R. Data entry double checks and fidelity monitoring may also be completed.

# Provisions to Monitor the Data to Ensure the Safety of Participants

Because this study Is a non-significant risk study, we will not require a DSMB.

# Provisions to Protect the Privacy Interests of Participants

Participants will be assigned unique study ID number when then are enrolled in the study, and all study data collected from that participant will be stored under that identifier. No identifying data will be stored with study data. The master key linking participants’ identities and study numbers will be stored separately under password protection and/or in locked cabinets, and will not be shared. Videos and photographs of study participants will not be shared without express written permission of the caregivers. Published results will use aggregate or de-identified data only.

# Compensation for Research-Related Injury

We will not provide any compensation for research related injuries.

# Economic Burden to Participants

We do not anticipate any significant economic burden to participants. Participants in the intervention group will receive the intervention in their home, at no charge. If participants do request in-clinic visits for the intervention, any parking expenses will be reimbursed. All participants will be reimbursed for each assessment time-point. They may also be provided with a parking voucher. Participants, or their insurance carrier, will be charged for non-study-related interventions or physician time during assessment visits. They will not be charged for any study-related developmental testing required as part of study assessments. For a typical visit to the Early Childhood Clinic, this will involve only the physician visits as the therapy assessments for the study are typical of what is normally done at a clinic visit. On rare occasions, a child may need an extra therapy assessment that is not part of the study (For example, the child may need to see a speech therapist in addition to the typical occupational therapy and physical therapy visits). They will not be charged for any study-related developmental testing required as part of study assessments. For participants who do not have access to necessary technology (e.g., smartphone, tablet, or computer) with which to watch videos, and who would otherwise qualify for the study and wish to participate, we may provide necessary resources to enable their participation, at the discretion of the PI.

# Consent Process

Consent will be required from the caregivers of the infant. Consent will take place while the infant is in the NICU or after discharge and may be completed in person, via telehealth, or telephone. Infants will be identified as potential participants in the study at approximately 33-34-weeks post-menstrual age (PMA) and may be consented up to approximately 48 weeks PMA.

# Non-English Speaking Participants

At least one of the infant’s caregivers (preferably the main caregiver) must speak and understand English, as the therapist(s) will need to be able to communicate effectively with the participant’s caregivers to provide the intervention, engage the caregiver(s), and document any barriers to implementation of the intervention or adverse events. In addition, caregivers will need to use Activity Playbook materials, which will be written/narrated in English, in order to provide the intervention. An inability of any caregiver to speak English is one of the listed exclusion criteria.

# Waiver or Alteration of Consent Process

No waivers of the consent process will be required. Because of the infant participants are under the age of 18, their consent is not required, and because of their age, their assent cannot be obtained. Consent will be obtained from biologic or adoptive parents or an individual legally authorized to consent on behalf of the child to general medical care.

***Process to Document Consent in Writing***

Parental permission will be obtained from one parent even if the other parent is alive, known, competent, reasonably available, and shares legal responsibility for the care and custody of the child.

Screening of potential study participants, those who decline, and those who consented and enrolled in the study, as well as a description of how consent was obtained, will be documented in EPIC. Study participants will be flagged in EPIC. Consent forms will be stored at the infant’s enrollment site, in REDCap, and in Study Tracker (as applicable).

*Setting:*

Participants will be recruited in the NICU at Lurie, Prentice, or UIC. Study staff will approach potential participants once the infant is medically stable, after obtaining permission from the child’s attending neonatologist. The research, which comprises a physical therapy intervention, will be conducted in the NICU until discharge, and then continued in the infant’s home. Assessments will be conducted in the clinic associated with the infant’s recruitment site. If necessary, intervention sessions or assessment sessions can be conducted at the infant’s home, or at another clinic site, depending on the participating family’s schedule or preference.

*Study Coordination:*

Study coordination will be performed by a Logistics & Management core, comprising site PIs from all participating study sites and the project PI, along with support staff from SRAlab. This core will provide the administrative, clinical, and technical expertise and leadership in the design and coordination, and implementation of this multi-site clinical trial. This core, led by the study PI, will be responsible for study monitoring at all sites and for coordinating participant recruitment, screening, enrollment and retention, for data and safety monitoring, data collection and analysis, adherence to the protocol-directed procedures and guidelines, and the prompt review, reporting and resolution of adverse events. External consultants will assist with protocol development and review study documents, but will not have access to any information on study participants.

### *Lead Coordinating Center*

SRAlab will provide the administrative and technical support for the study, and will coordinate subject recruitment, intervention delivery, and data collection across participating sites.

*Data Coordinating Center:*

SRAlab will act as the data coordinating center. We will establish Data Sharing Agreements with Lurie, Prentice, and UIC to enable access to their data as part of the study.

# Protected Health Information (PHI and HIPAA)

The research will involve collection of protected health information, including but not limited to participants’ name, birth date, parental information, NICU admission and discharge dates, address, information from infant and maternal medical records, and photographic/video images; caregiver’s telephone number, photographic/video images, and social security number (to enable study reimbursements). Language informing participants of the collection of this data and who may have access to it, as required by HIPAA. will be included on the consent form.

# Qualifications to Conduct Research and Resources Available

We estimate that up to 75 children per year from Prentice NICU will be eligible to participate in the study (>60 per year with birth weights less than 1500 grams and approximately 15-20 per year meeting neurological inclusion criteria). We estimate that up to 35 children from Lurie NICU will be eligible to participate (approximately 20 per year with birth weights less than 1500 grams and approximately 25 per year meeting neurological inclusion criteria). We expect to recruit approximately 35-40% of these infants to meet our recruitment goals. We expect to recruit approximately 13 babies per year from UIC.

Time (Calendar months) devoted to conducting and completing this research by Key Personnel:

PI: Raye-Ann deRegnier, MD, Yrs. 1-3: 2.4; Yrs. 4-5: 1.2; Yr. 6:0.6

Co-I Rick Lieber, PhD, Yrs. 1-6, 0.6

Co-I: Arun Jayaraman, PT, PhD, Yrs. 1-3: 3; Yrs. 4-6: 0.96

Co-I: Nishant Srinivasan, MD, Yrs. 1-3: 1.44; Yr. 4: 0.6; Yrs. 5-6: 0.36

Co-I Nicole Pouppirt, MD, Yrs. 1-3: 2.4; Yrs. 4-5: 1.2; Yr. 6:0.6

Co-I Cheryl Patrick, PT, MBA, Yr. 1: 1.8; Yrs. 2-5: 1.2; Yr. 6: 0.6

Co-I Megan O’Brien, PhD, Yrs. 1-3: 3; Yrs. 4-6: 6

Co-I Matthew Davis, MD, Yrs. 1-6: 0.6

Co-I Thomas Shanley, MD, Yrs. 1-6: 0.6

# multi-site research

Although not required by the Sponsor, we will establish a single-IRB for this study, with Lurie as the reviewing IRB (IRB of record) and UIC and NU(Prentice/SRAlab) as relying IRBs. We anticipate that the onboarding process for participating sites will occur concurrently with IRB review.

- No activities will occur at external sites until local IRB review is pursued or reliance agreements are fully executed.
- Any external site sign-offs or permissions will be acquired by external study teams in accordance with their local policies.
- IRB approval letters from external sites, documentation that IRB review at external sites is unnecessary, or fully executed reliance agreements will be provided when available with accompanying protocol updates.
- Non-compliance with the study protocol or applicable requirements will be reported in accordance with local policy

Potential subjects will be recruited at Lurie, Prentice, and UIC NICUs, and will be identified based on their medical history and examination including appropriate imaging. We will create flyers for distribution in the antenatal clinics and NICUs at participating sites (see attached documents).

Communication between sites will be accomplished by regular meetings of PIs, members of the logistics and management core, and project coordinators at each site. All sites will be provided with a copy of the current approved study protocol, consent document, and HIPAA authorization after each modification or revision, and all necessary approvals for each revision and continuing review will be obtained for each site before any changes are implemented.

All data will be maintained in REDCap, and data use agreements will be in place at each actively enrolling site to only allow access to data from that site. Data will be accessed by designated study staff at SRAlab, which is the data coordinating site for the study. Any necessary transmission of data will be achieved through HIPAA-approved email or cloud services. All participating sites will ensure confidentiality of personal health information and safety of data storage and transmission according to their individual institutional protocols, as required by the respective IRBs of record and state and Federal regulations and laws. Any and all instances of non-compliance with study protocols will be reported as required by respective institutional policies.

As the data coordinating center, SRAlab will be responsible for monitoring data fidelity, and will generate reports on a regular basis that will be shared with participating sites and reviewed at PI meetings to ensure data accuracy and completeness. SRAlab will also monitor adverse events and ensure all reporting requirements are met across sites. Meetings between PIs will be convened to address problems with study recruitment, and intervention implementation, and adverse events.

Interim results will be communicated with each site at PI meetings. Decisions on continuing of stopping the study will be made at each scheduled meeting or at additional meetings convened as necessary to address study findings or adverse events.

Data will be stored on REDCap indefinitely, and de-identified data as well as data analysis results will be provided to each site and made available to other investigators with necessary safeguards to ensure privacy of participants.
